# Regional Multi-Well Groundwater Level Forecasting in Sensor-Based Monitoring Networks Using a GCN-LSTM Framework

**DOI:** 10.3390/s26206588

**Published:** 2026-10-17

**Authors:** Kefan Chen, Songyi Dian, Xiaoying Li, Hongwei Fang

**Affiliations:** College of Electrical Engineering, Sichuan University, Chengdu 610065, China; 2024323035007@stu.scu.edu.cn (K.C.); scudiansy@scu.edu.cn (S.D.); xiaoyingli_lily@163.com (X.L.)

**Keywords:** groundwater level forecasting, GCN-LSTM, graph neural networks, sensor networks, groundwater monitoring, deep learning

## Abstract

**Highlights:**

**What are the main findings?**

- The proposed GCN-LSTM framework effectively forecasts groundwater levels across 56 monitoring wells by jointly modeling spatial relationships and temporal dependencies.
- Incorporating rainfall and water temperature as additional inputs improves forecasting performance and enhances the capability to capture groundwater level fluctuations.

**What are the implications of the main findings?**

- Graph-based deep learning provides a promising approach for exploiting spatial information in regional groundwater monitoring networks.
- The proposed framework supports intelligent groundwater monitoring and forecasting under different levels of sensor data availability.

**Abstract:**

Groundwater level forecasting is essential for groundwater monitoring and sustainable water resource management in urban regions. This study proposes a graph-based spatiotemporal forecasting framework for regional multi-well groundwater prediction using a Graph Convolutional Network Long Short-Term Memory (GCN-LSTM) model. Daily sensor-derived observations from 56 monitoring wells in Chengdu, China, collected from 2019 to 2023, are used to construct a graph representing inter-well spatial relationships. The GCN component captures spatial dependencies among wells, while the LSTM component models temporal variations in groundwater levels. Two data-availability scenarios are evaluated: a reduced-input setting and a multi-factor setting incorporating rainfall and water temperature. Results demonstrate that the proposed framework provides reliable groundwater level forecasts across multiple wells under both scenarios, and the inclusion of additional temporal variables improves the prediction of groundwater fluctuations. A network-wide controlled comparison under the S+W setting further shows that GCN-LSTM outperforms the conventional LSTM baseline across the 56-well monitoring network, supporting the contribution of inter-well spatial modeling to regional groundwater level forecasting. The findings highlight the potential of graph-based deep learning for regional groundwater forecasting and intelligent sensor-based monitoring networks.

## 1. Introduction

Groundwater is a fundamental component of urban water systems, supporting domestic and industrial supply, sustaining ecosystems, and buffering climate variability [1,2]. However, long-term intensive exploitation has led to widespread groundwater depletion and related environmental impacts such as land subsidence, aquifer degradation, and persistent declines in groundwater storage [3,4]. In parallel, rapid urbanization has substantially reshaped surface drainage patterns and intensified surface groundwater interactions. During extreme precipitation events, these interactions may trigger abrupt groundwater level fluctuations, increasing risks to underground infrastructure and complicating urban water-environment management [5,6,7,8]. To support sustainable groundwater governance, long-term and continuous monitoring programs have been established in many urban and regional aquifer systems [9]. The resulting observation records provide an essential data foundation for groundwater level forecasting, early warning, and management decision-making. Recent studies have further emphasized the value of wireless environmental sensor networks for groundwater-related monitoring and risk assessment, showing that end-to-end sensing pipelines can support continuous groundwater data acquisition, transmission, and environmental decision-making [10].

Despite the increased availability of monitoring data, reliable forecasting in real world urban settings remains challenging. First, groundwater time series are typically nonlinear and non-stationary due to coupled influences of hydrogeological conditions and time varying drivers [11,12]. Second, urban monitoring networks are often dense but irregularly distributed; groundwater dynamics at different wells exhibit strong spatial heterogeneity and complex inter-well dependencies, making regional prediction fundamentally a spatiotemporal learning problem rather than an independent single-well task [11,12,13]. Third, monitoring records frequently contain missing values, noise, and inconsistencies caused by sensor aging, maintenance interruptions, and measurement errors, which can degrade prediction reliability and limit model generalization [9,14]. These factors collectively motivate the development of robust spatiotemporal models that can (i) explicitly capture spatially distributed monitoring wells, (ii) learn temporal dynamics under nonlinearity and non-stationarity, and (iii) remain effective under realistic data-quality constraints.

A wide range of groundwater level prediction approaches have been explored, broadly falling into physically based models, statistical time-series models, traditional machine learning, and deep learning methods. Physically based groundwater models provide high interpretability by representing hydrological processes through conceptual or numerical formulations. For instance, MODFLOW has been widely adopted for groundwater flow simulation and management due to its ability to represent hydrogeological processes [15,16], while distributed hydrological models such as SHETRAN have been applied to groundwater related prediction by integrating spatial and hydro-climatological information [17]. Even though physically based models are valuable for scenario analysis, their practical application for long-term, regional multi-well prediction is often constrained by heavy requirements on parameterization, boundary condition specification, and calibration data, which may be difficult to satisfy in complex urban environments.

Statistical time-series models (e.g., ARIMA and related linear formulations) remain widely used baselines due to their simplicity and interpretability [18]. For example, Sakizadeh et al. [19] reported that time-series forecasting methods can achieve reasonable performance for site-specific groundwater level prediction under relatively stable conditions and sufficiently long observation records. However, classical statistical models are often sensitive to noise, missing values, and abnormal fluctuations [14]. Moreover, many existing groundwater forecasting studies focus primarily on temporal prediction at individual sites and do not explicitly model spatial interactions among wells [20], limiting their applicability to regional multi-well forecasting. In addition, as urban groundwater dynamics become increasingly nonlinear and influenced by multiple drivers, purely linear assumptions may not adequately reflect the coupled spatiotemporal processes.

Traditional machine learning methods, such as decision trees, support vector machines, random forests, and artificial neural networks, have been widely applied to groundwater prediction because of their nonlinear fitting capability and flexibility in feature engineering [21,22,23]. They can be computationally efficient and can incorporate heterogeneous covariates when domain knowledge is available. Under limited observation conditions, Mohammadi [24] combined MODFLOW-based simulation with artificial neural networks to estimate groundwater tables, illustrating the potential of hybrid strategies that integrate physically based modeling with data-driven prediction. Nevertheless, most traditional machine learning pipelines remain highly dependent on manual feature engineering and do not naturally encode inter-well spatial topology. As a result, they may struggle to capture high-dimensional spatiotemporal coupling patterns in regional multi-well monitoring networks.

In recent years, deep learning methods have demonstrated strong potential for hydrological time-series forecasting by learning hierarchical representations directly from data. Among deep learning models, Long Short-Term Memory (LSTM) networks are widely used to model nonlinear temporal dynamics and long-range dependencies [25,26]. In groundwater-related forecasting tasks, Wu et al. [27] developed a groundwater level modeling framework by combining wavelet transform with an LSTM-based data-driven model, demonstrating the effectiveness of LSTM-based methods in capturing temporal variations in hydrological sequences. To further enhance temporal modeling, attention mechanisms have been introduced to adaptively emphasize informative historical time steps and improve prediction accuracy in complex sequences; for example, Dai et al. [28] developed an attention-enhanced LSTM model for hydrological data prediction, showing advantages over baseline recurrent models and conventional approaches in water-level forecasting. These temporal deep learning models, however, are often formulated at the single-site level or treat spatial information through simplified aggregation, leaving the inter-well dependency structure underutilized in regional forecasting.

From a spatiotemporal perspective, groundwater systems are inherently spatiotemporal: heterogeneous aquifer properties and irregular well distributions induce spatial interactions, while groundwater dynamics evolve continuously over time [29,30,31]. Early attempts to incorporate spatial effects often relied on grid based spatial analysis or regression based formulations [32]. With advances in deep learning, Convolutional Neural Networks (CNNs) have been combined with temporal models to learn spatiotemporal representations in various forecasting tasks [33,34]. However, CNN based approaches generally assume grid-like spatial structures, which conflicts with real world groundwater monitoring layouts where wells form irregular, non-grid spatial topologies. This mismatch limits the ability of CNNs to represent meaningful spatial relationships among wells in regional groundwater prediction problems [33,34].

To address irregular spatial structures, Kipf and Welling [35] proposed Graph Convolutional Networks (GCNs), which provide a principled way to model data defined on graphs and enable representation learning over non-Euclidean topologies.

In spatiotemporal forecasting research, Zhao et al. [36] proposed the Temporal Graph Convolutional Network (T-GCN), which combines GCN-based spatial learning with recurrent units for temporal modeling and has achieved strong performance in traffic prediction problems. In groundwater studies, Chen et al. [37] further explored a spatiotemporal deep learning framework driven by attention to capture dynamic spatial and temporal dependencies in groundwater level prediction. These developments indicate that graph-based spatiotemporal learning is a promising direction for multi-well groundwater forecasting. Nevertheless, the application of such graph-based methods to regional scale multi-well groundwater level prediction remains limited. Key barriers include realistic data quality issues, heterogeneous feature availability across cities, and the need for scalable evaluation under different data availability scenarios that mirror practical urban water management settings.

Recent IoT-based water-level monitoring studies have shown that the integration of water-level sensors with temperature and humidity sensing, communication modules, data storage, and cloud-based processing layers can improve real-time water resource management and generate structured data streams for subsequent forecasting models [38]. Sensor-based forecasting systems have further incorporated multi-source environmental observations, such as rainfall, temperature, humidity, pressure, and water-level measurements, into learning-based prediction frameworks for real-time hydrological risk assessment [39]. These studies suggest that groundwater and water-level monitoring networks are evolving from passive observation systems toward data-driven sensing platforms that can support forecasting, risk assessment, and early warning.

Motivated by the above limitations, this study focuses on regional multi-well groundwater level forecasting in Chengdu, China, using daily observations from 56 state-owned monitoring wells collected between 2019 and 2023. Unlike conventional single-well prediction tasks, the problem is formulated as a regional spatiotemporal forecasting task in which groundwater dynamics are jointly influenced by regional monitoring layouts, heterogeneous well attributes, and time-varying hydro-meteorological factors. To better reflect practical monitoring conditions in urban groundwater systems, the study considers not only prediction accuracy but also model applicability under different levels of data availability. Accordingly, two forecasting scenarios are designed. The first scenario uses only groundwater level data and spatial attributes, while the second additionally incorporates rainfall and water temperature. This design makes it possible to evaluate the proposed framework under both simplified and information-rich conditions, and enhances its practical relevance for groundwater monitoring networks with different observational capacities.

To address this problem, a spatiotemporal forecasting framework based on GCN and LSTM is developed. In the proposed method, observation wells are represented as nodes in a graph, and inter-well relationships are characterized through spatial similarity and attribute similarity so that spatial dependence within the monitoring network can be explicitly modeled. The GCN component is used to learn spatial dependence and neighborhood interactions among wells, while the LSTM component captures temporal evolution and long-range dependencies in groundwater level sequences. By combining graph-based spatial learning with temporal sequence modeling, the proposed framework is expected to represent the coupled spatiotemporal behavior of regional groundwater systems more effectively than models that rely only on temporal dynamics or simplified spatial inputs.

The main contributions of this work are summarized as follows:

1. A graph-based spatiotemporal forecasting framework for regional multi-well groundwater prediction: A GCN-LSTM architecture is developed to jointly model inter-well spatial dependence and temporal groundwater dynamics in a sensor-based groundwater monitoring network.
2. A graph representation strategy for multi-well monitoring networks: Observation wells are represented as graph nodes, and inter-well relationships are constructed using both spatial proximity and well attribute similarity, enabling the model to capture heterogeneous spatial interactions that are difficult to represent using conventional sequence models.
3. A practical evaluation under different data availability scenarios: Two forecasting settings are established, including one based only on groundwater level data and another that further incorporates rainfall and water temperature, in order to assess the robustness and applicability of the framework under realistic monitoring conditions.
4. Validation using observations from a regional monitoring network: Using sensor-derived daily data from 56 wells in Chengdu over a five-year period, the proposed framework is shown to provide reliable and stable multi-well groundwater level forecasts, demonstrating its potential value for regional groundwater monitoring, forecasting, and early warning.

The remainder of this paper is organized as follows. Section 2 describes the study area, monitoring network, sensor-derived data, data preprocessing procedures, graph construction strategy, and GCN-LSTM forecasting framework. Section 3 presents the experimental design and forecasting results under different input settings. Section 4 discusses the main findings, the role of spatial dependence and sensor-derived temporal variables, and the practical implications for groundwater monitoring and early warning. Section 5 concludes the paper and outlines future research directions.

## 2. Materials and Methods

This section describes the materials and methods used for regional multi-well groundwater level forecasting. It first introduces the Chengdu study area and the monitoring network, followed by the sensor-derived groundwater level, water-temperature, and rainfall datasets. The data preprocessing procedures, graph construction strategy, and GCN-LSTM forecasting framework are then presented in detail.

### 2.1. Study Area

The study area is located in Chengdu, Sichuan Province, China. The region is mainly characterized by plains and hills, with elevations in the main urban area generally ranging from about 500 to 600 m above sea level, while the surrounding areas extend to higher elevations. Chengdu exhibits complex geological and hydrogeological conditions, with substantial spatial heterogeneity in terrain, subsurface structure, and groundwater response. These characteristics contribute to the complexity of groundwater dynamics across the urban monitoring network.

Groundwater levels in Chengdu are influenced by multiple interacting factors, including rainfall infiltration, surface water inputs, geological conditions, and human activities. Short-duration intense rainfall can enhance infiltration and cause noticeable groundwater level rises, while the effects of surface water inputs may weaken with increasing distance from water bodies and may also exhibit a lagged response. In addition, differences in topography and subsurface conditions can influence both the rate and magnitude of groundwater level variation. Under rapid urbanization, these hydrogeological processes become more complex, making Chengdu a representative case for regional multi-well groundwater level forecasting in urban environments.

### 2.2. Monitoring Network and Sensor-Derived Data

**Figure 1:**
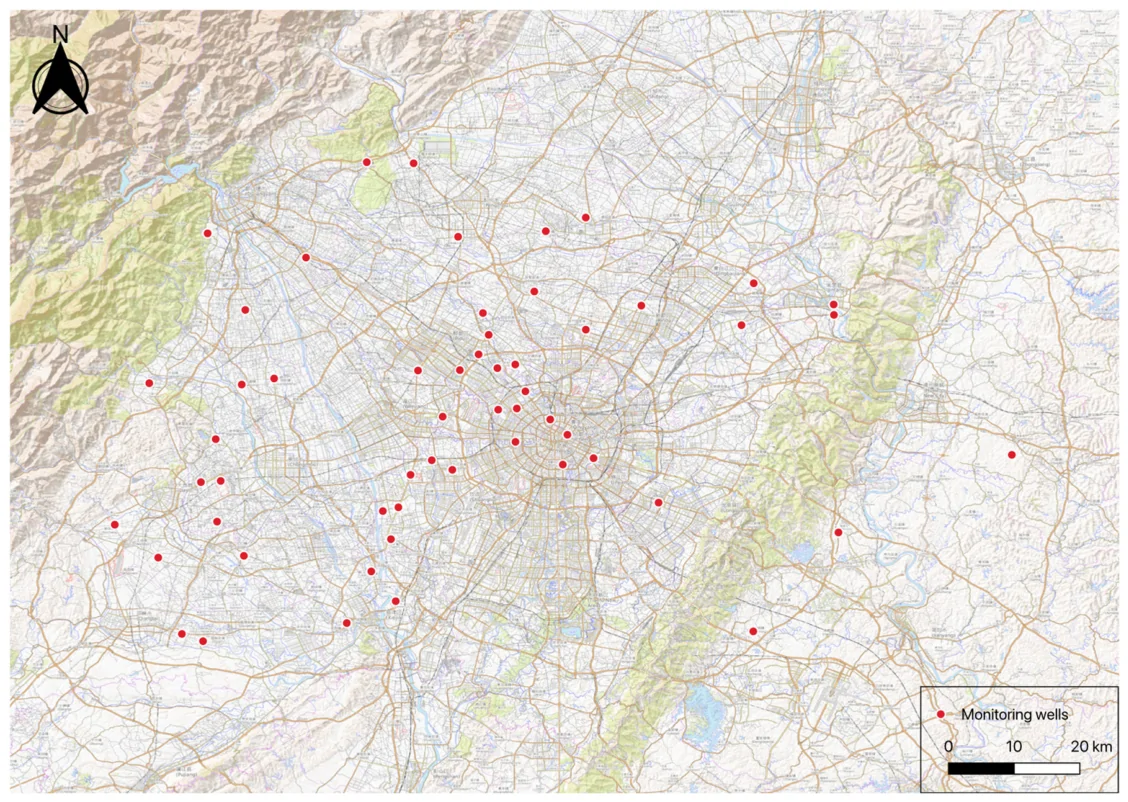
Spatial Distribution of 56 Groundwater Level Observation Wells.

The dataset used in this study is sourced from the China Institute of Geological Environment Monitoring [40]. It consists of site data from the National Groundwater Monitoring Project in Chengdu, comprising a total of 56 monitoring stations. The specific distribution of these stations is shown in Figure 1.

Groundwater levels are influenced by a combination of various spatial features, among which latitude and longitude, wellhead elevation, and well depth are key factors. Summary statistics for the first 10 observation wells are shown in Table 1 (the complete table is provided in Appendix A.1). Firstly, latitude and longitude determine the regional climate and topography, which in turn affect groundwater recharge and consumption. Within the same latitude and longitude range, variations in terrain elevation and surface cover significantly impact the formation, flow, and storage of groundwater. Secondly, the wellhead elevation, representing the ground surface elevation at the monitoring well, determines the vertical distance between the wellhead and the groundwater level. Finally, the well depth directly affects the depth of the aquifer reached by the well. A greater well depth indicates that the well has penetrated deeper groundwater layers, while a shallower well depth suggests a relatively shallow groundwater table. These spatial features interact and collectively shape the spatial distribution patterns of groundwater levels, playing a critical role in groundwater resource utilization and water management.

The dataset used in this study includes groundwater level data from a total of 56 observation wells in Chengdu, covering the period from 1 January 2019, to 31 December 2023, with a total of 1826 records. A portion of the groundwater level data from the study area is shown in Table 2 (the complete table is provided in Appendix A.2). The table indicates that wells well-7, well-17, well-24, well-42, and well-52 have relatively large ranges and standard deviations, suggesting that the groundwater levels at these wells are more variable.

In addition to groundwater levels, the dataset includes rainfall observations for Chengdu and water-temperature records for each of the 56 wells over the study period. A common city-average rainfall series was used for all wells, whereas water temperature was represented by the individual record from each well. The shared rainfall input represents regional variability but does not resolve local differences in precipitation across the monitoring network.

### 2.3. Sensor Specifications

The groundwater level observations used in this study were obtained from automatic liquid-level monitoring instruments installed at observation wells. The level sensing unit was based on a submersible hydrostatic liquid-level transmitter (model: CHZB-PLG001, manufacturer: Hangzheng Technology Co., Ltd., Wuxi, China), which converts water pressure into water-level measurements. The sensor adopts a fully sealed isolation diaphragm structure with a stainless-steel membrane and a high-stability pressure-sensitive chip, enabling long-term operation in humid and submerged environments. The sensing system supports RS485/Modbus digital communication and can be integrated with a remote terminal unit (RTU) for data acquisition and transmission.

According to the available technical specification, the liquid-level sensor has a measurement range of 0−15 m, an accuracy of 0.1%, and an IP68 protection rating. The sensor is powered by 12/24 VDC, supports RS485/Modbus communication, and is compatible with liquids in contact with 316L stainless steel. The operating temperature ranges from −20 °C to 65 °C for the environment and from −20 °C to 85 °C for the measured medium. The sensor is installed by direct submersion, with cable output for signal transmission. These specifications indicate that the monitoring instruments are suitable for long-term groundwater level data acquisition under field conditions.

**Figure 2:**
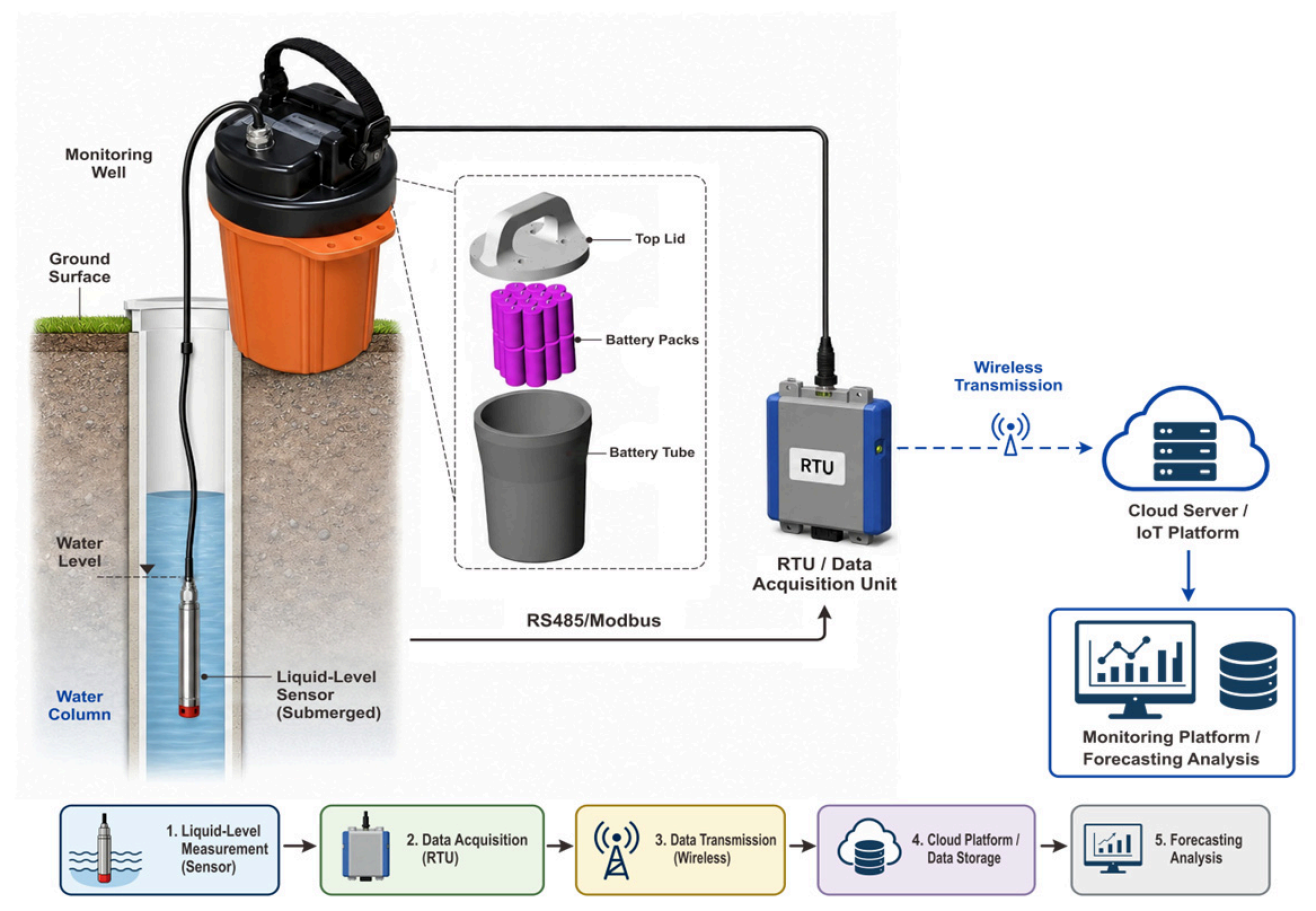
Schematic diagram of the liquid-level sensing and data acquisition system.

The monitoring system also includes an RTU module for data reading, local control, and remote transmission. The RTU supports RS485 and RS232 interfaces, configurable baud rates, low-power operation, and remote parameter configuration. The data acquisition and transmission frequencies can be adjusted according to monitoring requirements. Communication between the terminal device and the data platform can be implemented through MQTT, while the platform can retrieve data through HTTP. Figure 2 illustrates the liquid-level sensing and data acquisition configuration used for water-level monitoring, including the submersible liquid-level sensor, RTU, communication interface, and remote data transmission process. The main components of the monitoring system and their technical specifications are summarized in Table 3.

### 2.4. Data Preprocessing

To prevent potential data leakage, the chronological training and testing split was established before data-dependent preprocessing. The Random Forest imputation models and Min-Max normalization parameters were fitted exclusively using the training data and subsequently applied to the testing data. Wavelet denoising was performed without using observations beyond the corresponding prediction time, ensuring that future information was not introduced into the prediction inputs.

Due to the diverse regional geological features of Chengdu characterized by complex geological structures and significant topographical variations, groundwater level fluctuations exhibit considerable complexity. Groundwater monitoring records may exhibit nonlinearity, non-stationarity, noise, and missing observations because of interacting hydrogeological, climatic, and anthropogenic influences. Data preprocessing was therefore performed before model construction to improve data consistency and support stable forecasting [41,42].

#### 2.4.1. Missing Value Handling

The groundwater monitoring datasets may contain missing observations due to sensor failures, communication interruptions, or maintenance activities. To ensure temporal continuity before model training, missing records in groundwater level and water temperature observations were identified and reconstructed.

**Figure 3:**
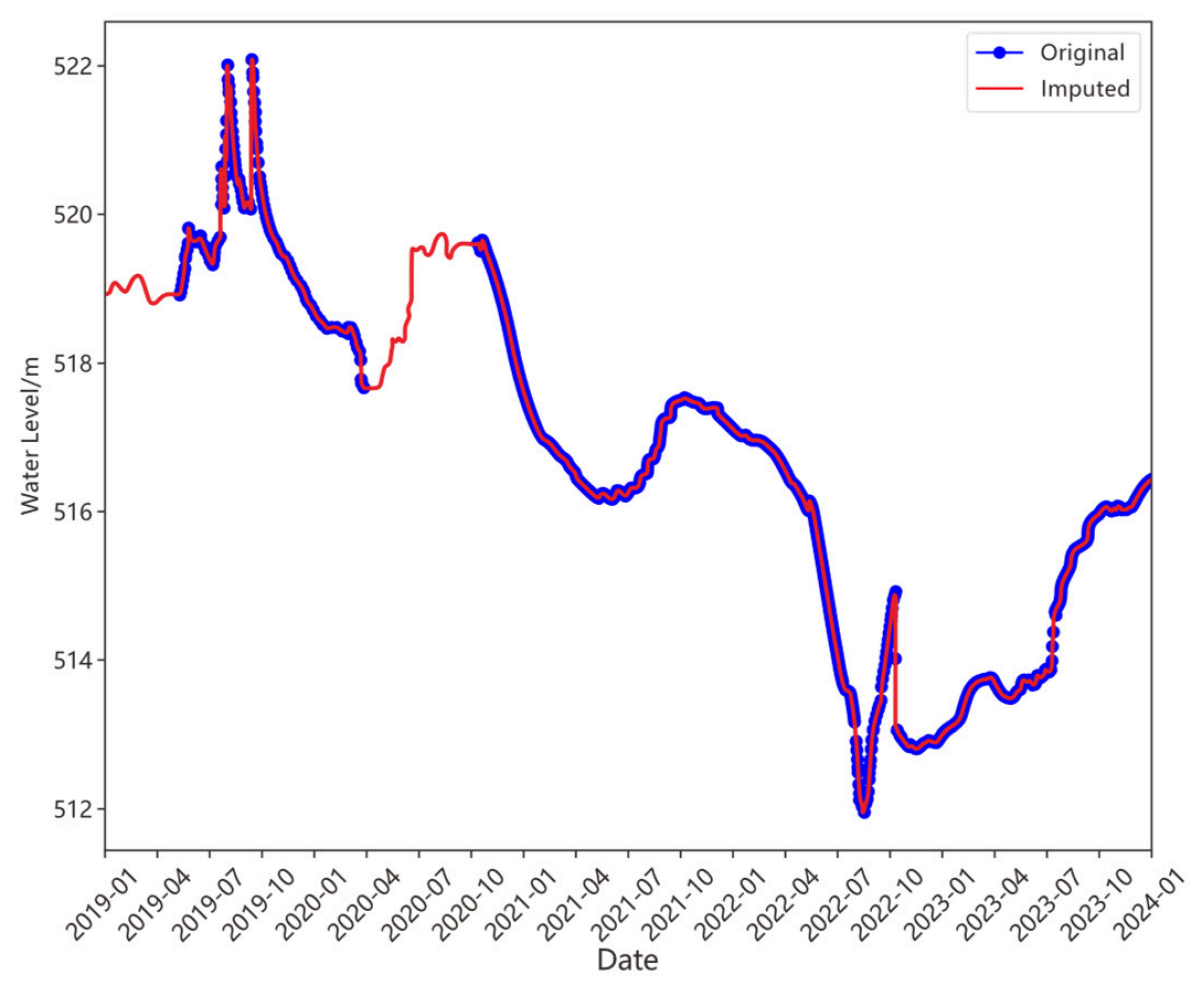
Random Forest Imputation Result for Missing Values.

Missing observations were first identified and then reconstructed using a random forest-based imputation method. Random Forest was selected because it can capture nonlinear relationships among variables and has been demonstrated to be effective for missing data reconstruction [43]. Figure 3 presents an example of the reconstructed groundwater level series.

Before imputation, 20,809 of the 102,256 expected groundwater level observations across the 56 monitoring wells were missing, corresponding to an overall missing rate of 20.35%. The missing rate varied substantially among wells, ranging from 0.55% to 56.63%, with a median of approximately 17.96%. The difficulty of reconstruction therefore differed among wells depending on both the amount and temporal distribution of missing observations. Wells with sparse or isolated missing values were generally less challenging than those containing larger missing proportions or longer continuous gaps.

After imputation, all missing groundwater level records were reconstructed while the originally observed values were retained unchanged. The reconstructed values were also checked against the observed value ranges of the corresponding wells to avoid unrealistic extreme values.

#### 2.4.2. Data Denoising

**Figure 4:**
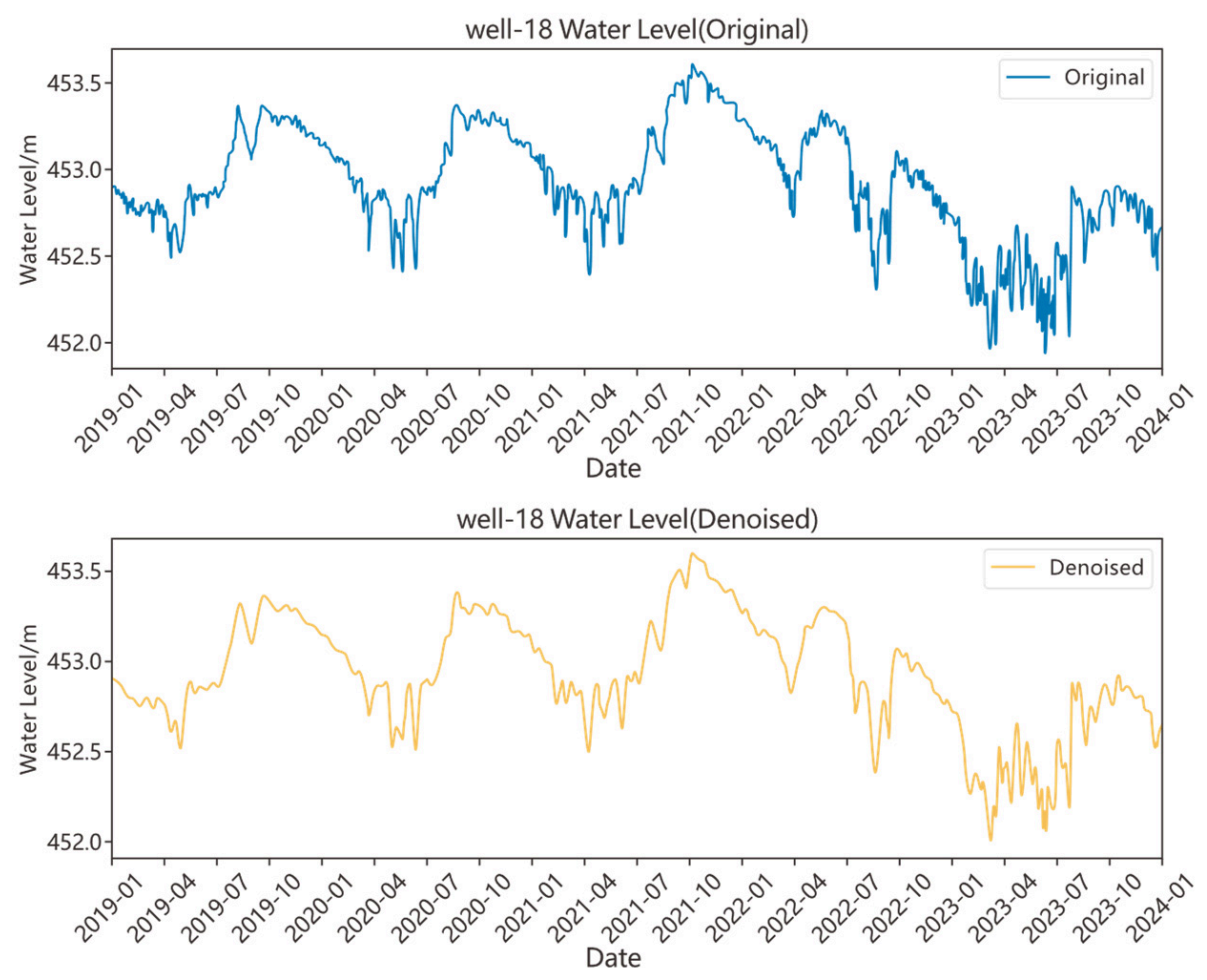
Comparison of Wavelet Denoising Results for Well-18.

To reduce the interference of noise in groundwater level prediction for Chengdu, wavelet transform techniques are applied to denoise the raw groundwater level data. Wavelet transform is a widely used signal processing method for noise suppression and feature extraction in non-stationary hydrological time series, and has been proven effective in enhancing groundwater level forecasting performance [44]. Figure 4 presents the comparison of groundwater level data before and after denoising for the representative observation well-18.

As clearly shown in the figures, the original data contains noise signals such as spikes and fine fluctuations. After applying wavelet transform for denoising, these high-frequency noise components are effectively removed, making the true signal more distinct and recognizable. This enhances the accuracy of groundwater level prediction in Chengdu, thereby supporting more effective management and protection of the city’s groundwater resources.

#### 2.4.3. Data Normalization

Due to differences in data sources and measurement methods for groundwater level features in Chengdu, the value ranges of groundwater level data vary significantly. Therefore, normalization is required before model training. The time-series variables were normalized using min–max scaling, with scaling parameters estimated separately for each variable at each well using only the training data. The fitted parameters were then applied unchanged to the training, validation, and test sets. This transformation maps the training-data range to [0, 1], while transformed validation and test values may fall outside this interval when they exceed the corresponding training-data range.

#### 2.4.4. Rainfall Data Downsampling

Rainfall was recorded at 3-h intervals, at 02:00, 05:00, 08:00, 11:00, 14:00, 17:00, 20:00, and 23:00. The eight 3-h precipitation totals for each day were summed to obtain daily rainfall, matching the daily resolution of the groundwater level and water-temperature observations.

**Figure 5:**
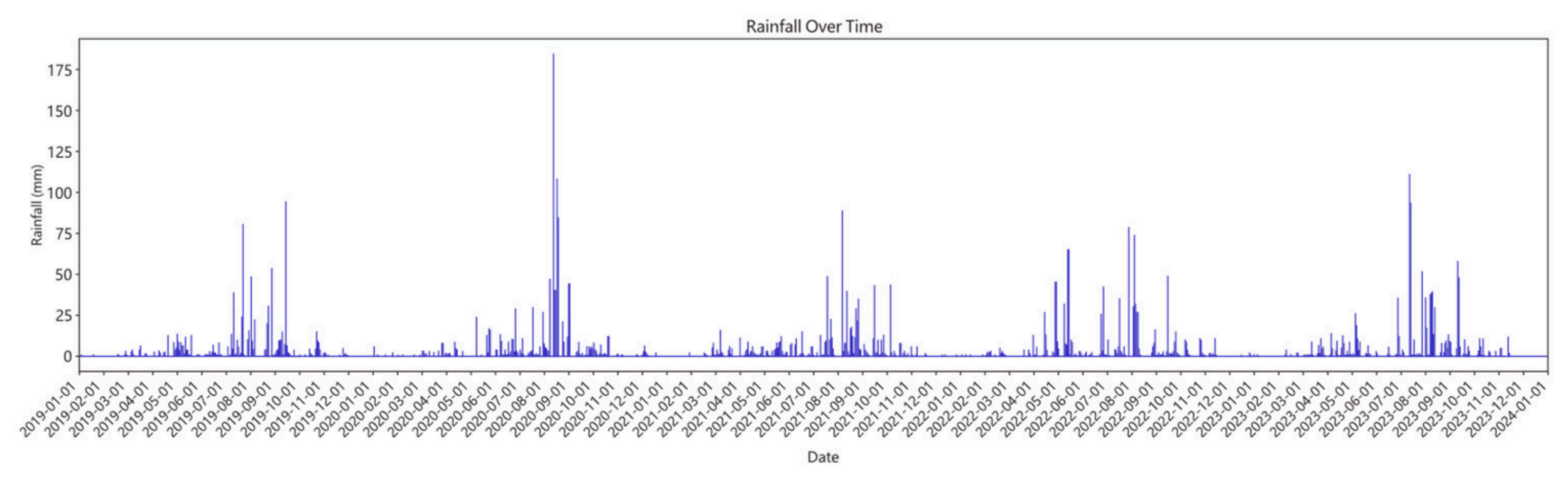
Rainfall Data Visualization from 2019 to 2023.

Figure 5 shows the rainfall series over the study period, with generally higher precipitation in summer. The same rainfall series was used as an input for all 56 wells. This shared input represents regional rainfall conditions but does not resolve local differences in precipitation across the monitoring network or imply identical groundwater responses among wells.

**Figure 6:**
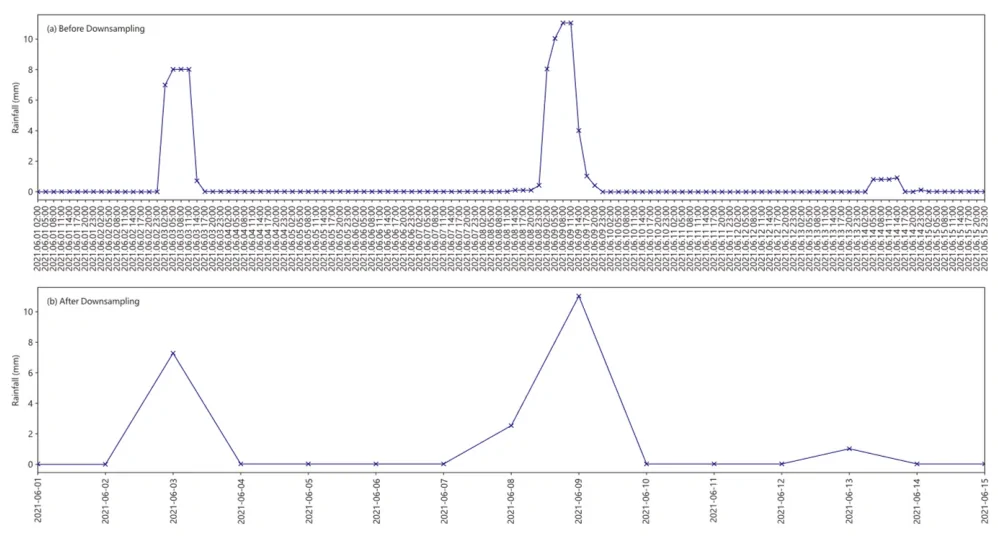
Comparison of rainfall series before and after temporal downsampling: (**a**) original rainfall series at 3-h intervals; (**b**) daily rainfall series after downsampling.

Figure 6 compares the original 3-h rainfall series with the daily series obtained after temporal downsampling for 1–15 June 2021. The downsampled series broadly preserves the timing of the main rainfall events while representing rainfall variation at a coarser temporal resolution. This step aligns the temporal resolution of the rainfall input with that of the daily groundwater level series used for subsequent modeling.

### 2.5. Graph Construction for Monitoring Networks

In this section, a GCN–LSTM framework is presented for regional multi-well groundwater level forecasting, aiming to capture inter-well spatial dependencies and temporal groundwater dynamics in a unified manner. The method first encodes the monitoring wells into a graph representation, where inter-well relationships are characterized by a distance-based spatial similarity matrix and an attribute similarity matrix derived from well location and static attributes. Based on this spatial representation, a coupled GCN–LSTM network is constructed, in which the GCN learns spatial features via neighborhood aggregation and the LSTM models temporal evolution for multi-well forecasting.

#### 2.5.1. Graph Representation of Observation Wells

Graph networks provide a structured framework for representing entities and their pairwise relationships through nodes and edges [45]. Accordingly, the 56 observation wells are modeled as nodes, while the relationships between wells are represented as weighted edges. Each node corresponds to one monitoring well, and the edge weight characterizes the strength of the spatial relationship between two wells. In this study, inter-well relationships are characterized using both geographic proximity and static well-attribute similarity. The static attributes include longitude, latitude, wellhead elevation, and well depth. This graph representation allows the monitoring network to be incorporated into the GCN component, where groundwater level information can be aggregated from related wells according to their spatial and attribute-based connections.

#### 2.5.2. Spatial Similarity Matrix

Based on the spatial distribution of the 56 groundwater level observation wells in Chengdu, this study uses Euclidean distance to represent the spatial similarity between wells. It is worth noting that the horizontal distance *d* between wells is significantly greater than the elevation difference *h*, so the angle between the line connecting two wells and the horizontal plane is considered negligible. To emphasize the impact of spatial distance on similarity, the inverse of the squared distance between wells is used. The spatial weight matrix *W_ij_* is calculated as follows:

(1) $$W_{\mathrm{ij}}=\left\{\begin{array}{c} \frac{1}{d^{2}},i\neq j\\ 0,i=j \end{array}\right.$$

#### 2.5.3. Attribute Similarity Matrix

When examining the groundwater level data of a single well in isolation, it becomes apparent that its groundwater level is not only influenced by the levels of neighboring wells, but can also be partially explained by its own spatial attributes, including longitude (A), latitude (B), wellhead elevation (C), and well depth (D). Longitude and latitude primarily characterize the horizontal geographic relationships among monitoring wells, whereas wellhead elevation and well depth provide complementary vertical and structural information. Recent graph-based groundwater forecasting studies have similarly shown that inter-well dependencies may arise from multiple spatial relationships, including physical proximity and shared environmental characteristics [46]. Longitude and latitude therefore have a more direct influence on the spatial connectivity of the graph, while wellhead elevation and well depth help distinguish wells with similar geographic locations but different vertical or structural characteristics. Together, these two groups of features characterize inter-well relationships from complementary spatial perspectives. For instance, when a particular observation well exhibits a high groundwater level, this may not only be due to a higher volume of groundwater, but also because of its higher elevation. Before calculating the attribute similarity, longitude, latitude, wellhead elevation, and well depth were independently normalized to the [0, 1] range using min–max normalization [47], a commonly used feature scaling method for reducing differences in numerical scales among attributes.

In this study, cosine similarity is used to measure the attribute similarity between any two observation wells. Suppose observation well *X_i_* has an attribute vector ${\vec{v}}_{1}=(A_{1},B_{1},C_{1},D_{1})$, and observation well *X_j_* has a vector ${\vec{v}}_{2}=(A_{2},B_{2},C_{2},D_{2})$, then the attribute similarity between the two wells can be represented by the cosine of the angle between the two vectors:

(2) $$\cos\theta_{\left\langle i,j\right\rangle }=\frac{{\vec{v}}_{i}\cdot {\vec{v}}_{j}}{\left\|{\vec{v}}_{i}\|\cdot \|{\vec{v}}_{j}\right\|}=\frac{A_{i}A_{j}+B_{i}B_{j}+C_{i}C_{j}+D_{i}D_{j}}{\sqrt{A_{i}^{2}+B_{i}^{2}+C_{i}^{2}+D_{i}^{2}}\cdot \sqrt{A_{j}^{2}+B_{j}^{2}+C_{j}^{2}+D_{j}^{2}}}$$

Similarly, the attribute similarity values between all 56 observation wells can be obtained. Therefore, in this study, the attribute similarity matrix is defined as C_ij_ = cosθ_⟨i,j⟩_, where i and j represent the attribute vectors of any two observation wells.

#### 2.5.4. Final Adjacency Matrix

Based on the spatial similarity matrix and the attribute similarity matrix, the final edge weight between observation wells *i* and *j* is defined as the product of their spatial similarity *W_ij_* and attribute similarity *C_ij_*. The multiplicative formulation is used to represent a joint constraint between geographic proximity and attribute similarity. A strong edge is therefore assigned only when two wells are both spatially close and similar in their static characteristics. Therefore, the spatial aggregation of groundwater level information can be expressed as:

(3) $$X_{i}=\sum_{j\in N(i)}W_{\mathrm{ij}}C_{\mathrm{ij}}X_{j}$$

where *X_i_* denotes the groundwater level feature of well *i*, *X_j_* denotes the groundwater level feature of neighboring well *j*, *W_ij_* represents the distance-based spatial similarity, and *C_ij_* represents the attribute similarity. In this formulation, *W_ij_C_ij_* corresponds to the final adjacency weight between wells *i* and *j*. The constructed adjacency matrix is then used as the graph input to the GCN component for spatial feature aggregation.

### 2.6. GCN-LSTM Forecasting Framework

**Figure 7:**
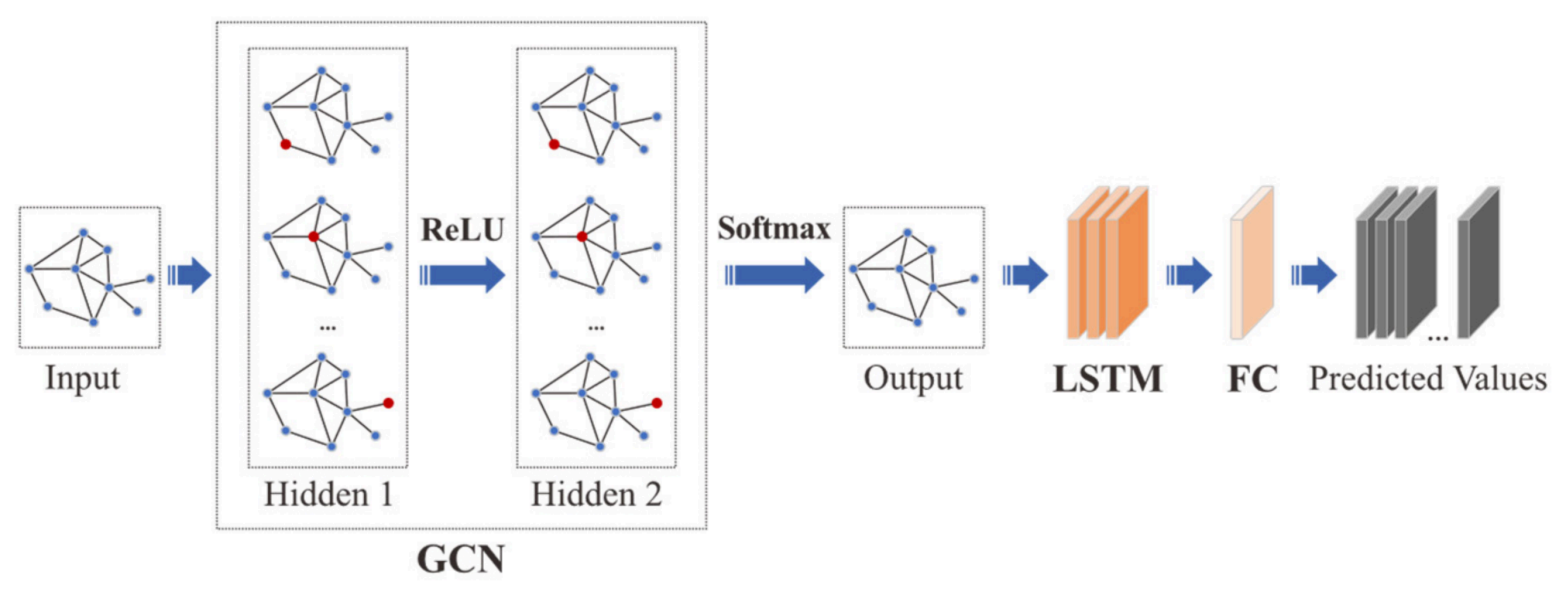
GCN-LSTM Architecture Diagram.

To address the challenges in regional multi-well groundwater level prediction, such as significant spatial distribution differences among wells and strong inter-well correlations, a GCN-LSTM based spatiotemporal prediction algorithm is proposed. This algorithm considers not only the historical groundwater level data from multiple wells but also the influence of spatial distribution differences on prediction accuracy. First, the spatial location and attribute features of the observation wells are organized into a graph structure using an adjacency matrix. Then, the GCN is employed to extract spatial dependencies from the graph-structured data, while the temporal features of groundwater levels are captured to model spatiotemporal correlations. Finally, the model simultaneously outputs groundwater level predictions for multiple wells, enabling multi-point and regional-scale forecasting. The detailed GCN-LSTM network structure is illustrated in Figure 7.

## 3. Results

This section presents the experimental design and forecasting results of the proposed GCN-LSTM framework. To evaluate its applicability under different monitoring conditions, two input settings are considered: one using spatial features and historical groundwater level records only, and the other further incorporating rainfall and water temperature as sensor-derived temporal variables. The implementation details, experimental settings, data splitting strategy, and evaluation metrics are first described, followed by a comparative analysis of the prediction performance under the two input settings.

### 3.1. Implementation Details

The proposed GCN-LSTM framework was implemented using PyTorch version 2.6.0. The spatial encoder consisted of two graph convolutional layers, where node features were transformed into a 128-dimensional spatial representation through an intermediate hidden layer with 100 neurons. The temporal dependency was subsequently modeled using a two-layer LSTM network with 128 hidden units and a dropout rate of 0.5. The final groundwater level prediction was generated through fully connected layers with dimensions of 128–64–1.

During model training, the Adam optimizer was adopted with a learning rate of 0.006 and a weight decay coefficient of 10^−4^. The mean squared error (MSE) was used as the optimization objective. The model was trained for 200 epochs with a batch size of 128. A five-day historical sequence was used as temporal input for next-day groundwater level prediction. This short-term window was selected to capture the recent evolution of groundwater levels while maintaining a compact temporal input and limiting unnecessary historical redundancy. The same training configuration was applied consistently across the corresponding controlled experiments to ensure a fair comparison between different input scenarios and model architectures. For the network-wide comparison between GCN-LSTM and LSTM, each model was independently trained using five random seeds, and the results are reported as the mean ± standard deviation.

### 3.2. Experimental Settings

The dataset consists of continuous five-year observation records from 56 groundwater monitoring wells in Chengdu, spanning from 1 January 2019 to 31 December 2023, totaling 1826 days. Both groundwater level and water temperature time-series data are recorded once per day, while rainfall data is recorded eight times per day. The rainfall data were converted to a daily series using the procedure described in Section 2.4.4 to match the temporal resolution of the groundwater level and water-temperature observations. For the spatial features (longitude, latitude, wellhead elevation, and well depth) that do not change over time, the spatial feature dataset has a size of 56 × 4. Based on the longitude and latitude of the 56 observation wells, an adjacency matrix *A_t_* is constructed, consisting of a 56 × 56 spatial weight matrix *W_ij_* and a 56 × 56 attribute similarity matrix *C_ij_*. This adjacency matrix expresses the spatial and attribute relationships among the observation wells.

In this research, groundwater level prediction is validated and analyzed using the GCN-LSTM network on two datasets of different scales. The first dataset considers only spatial features and groundwater level data, while the second includes spatial features along with three types of temporal features for prediction (water temperature, rainfall, and groundwater level). Two separate datasets were constructed:

- A groundwater level dataset over 1826 days, with a size of 1826 × 56.
- A combined dataset containing water temperature, rainfall, and groundwater level features, with a size of 1826 × (56 × 3).

Both datasets follow the same data splitting strategy for training and testing: a total of 80% of the data (1460 days) is used for training, and the remaining 20% (366 days) is used for testing. An examination of the original groundwater level records revealed that 19,956 of the 20,496 possible well-day observations (97.37%) in the specified 366-day testing period were originally observed, while the remaining 540 observations (2.63%) required imputation. These statistics provide additional transparency regarding the completeness of the testing dataset. Well-by-well statistics on missing observations over the full study period are provided in Appendix A.4 (Table A4).

To further evaluate the contribution of graph-based spatial modeling, an additional network-wide controlled comparison was conducted between the proposed GCN-LSTM and a conventional LSTM under the S+W setting. The S+W setting was selected because it provides the more parsimonious input configuration and allows the contribution of inter-well spatial modeling to be evaluated without the additional influence of rainfall and water-temperature variables. Both models used the same groundwater level observations, five-day historical input window, chronological training and testing split, normalization procedure, optimization settings, and evaluation data. The principal difference between the two models is that GCN-LSTM explicitly incorporates the graph representation of inter-well relationships before temporal modeling, whereas the conventional LSTM models the temporal evolution without graph-based inter-well information. Each model was trained using five random seeds, and network-wide performance was evaluated across all 56 monitoring wells using MAE, RMSE, and R^2^.

In addition to the network-wide evaluation of the proposed framework, a supplementary single-well benchmark was conducted for well-56. This benchmark compares GCN-LSTM with conventional recurrent and attention-based forecasting models for which results were available under the same target-well setting.

### 3.3. Prediction Considering Only Spatial Features and Groundwater Level

**Figure 8:**
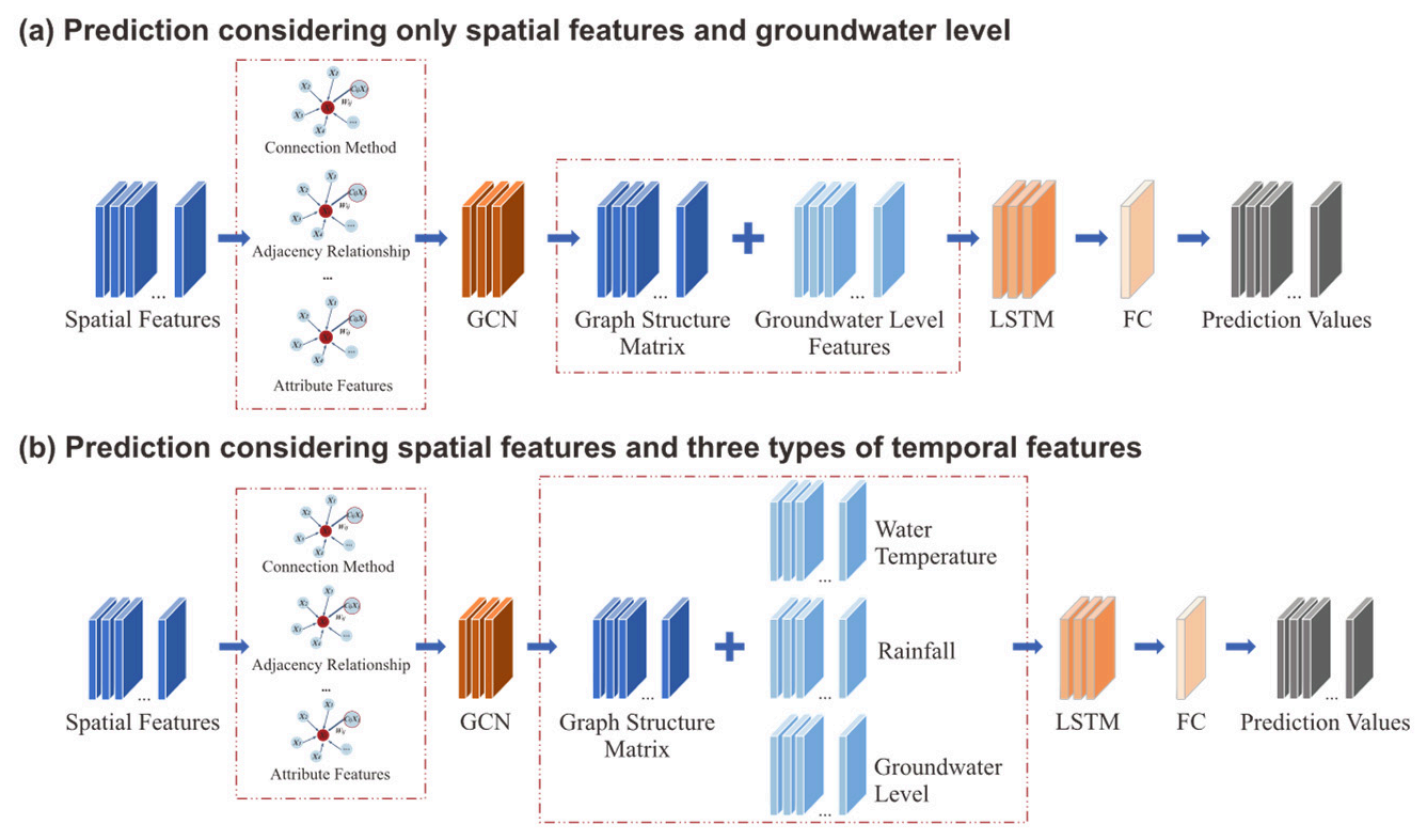
Model architectures under the two input settings: (**a**) S + W, using spatial attributes and historical groundwater levels; (**b**) S + T, using spatial attributes, historical groundwater levels, rainfall, and water temperature.

This scenario focuses more on the fundamental factors in groundwater level prediction. It primarily considers the spatial distribution characteristics of groundwater levels and the spatial correlations among different observation wells. This setting addresses forecasting within an existing monitoring network when historical groundwater level observations and well spatial attributes are available but rainfall and water-temperature inputs are unavailable. It does not evaluate prediction at wells excluded from training or at unmonitored locations. Using the GCN-LSTM model to predict groundwater levels across multiple wells in a region can still yield satisfactory results. However, this scenario neglects the influence of other temporal factors on groundwater levels. Therefore, its performance may be limited in real-world scenarios where seasonal variations, climate change, and other temporal dynamics play a significant role. The network structure for predictions considering only spatial features and groundwater level data is shown in Figure 8a.

### 3.4. Prediction Considering Spatial Features and Three Types of Temporal Features

Compared to the prediction scenario that considers only spatial features and groundwater level data, the scenario incorporating spatial features along with three types of temporal features provides a more comprehensive approach. In this setting, in addition to the spatial distribution characteristics of groundwater levels, water temperature and rainfall are also introduced, as both factors significantly influence the groundwater levels across multiple wells in a given region. This scenario is particularly suitable for modeling complex groundwater systems, especially in areas where groundwater dynamics are strongly affected by seasonal patterns, climate change, and other temporal influences. In some regions with stricter municipal management, a greater number of sensors and more extensive historical data are available, resulting in richer data resources for analysis. In such data-rich environments, incorporating multiple temporal features allows for more accurate capture of the spatiotemporal variations in groundwater levels, thereby improving prediction precision and reliability. The network architecture for prediction considering spatial features and three temporal features is shown in Figure 8b.

### 3.5. Model Evaluation Results Across All Monitoring Wells

**Figure 9:**
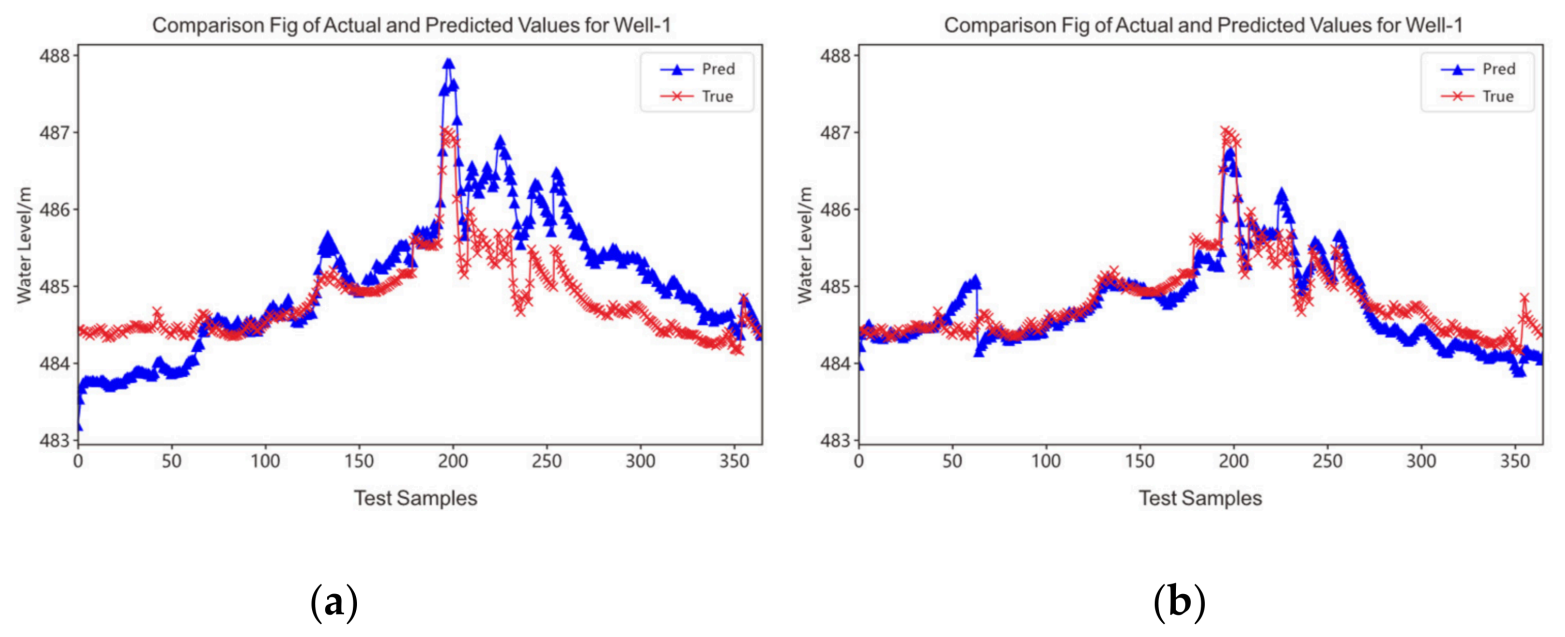
Prediction results for well-1 under two input settings. (**a**) Spatial features + groundwater level. (**b**) Spatial features + groundwater level + rainfall and water temperature.

**Figure 10:**
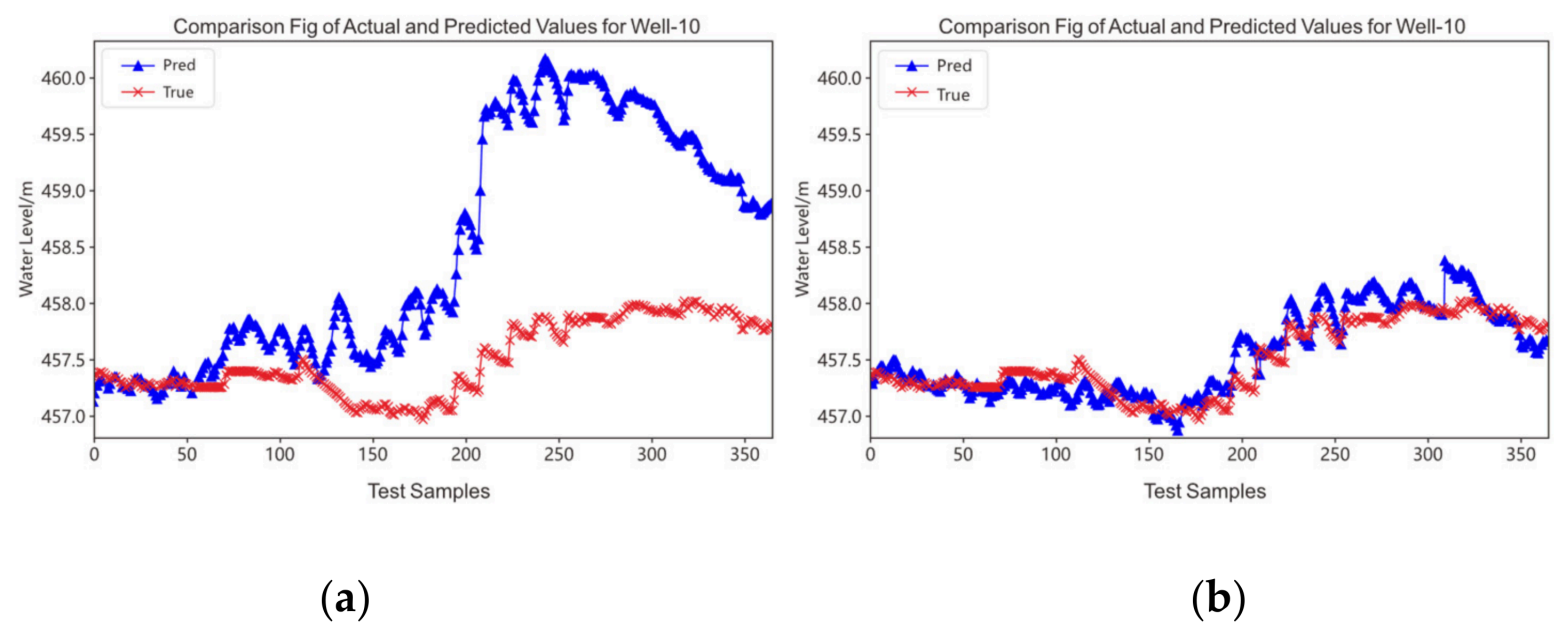
Prediction results for well-10 under two input settings. (**a**) Spatial features + groundwater level. (**b**) Spatial features + groundwater level + rainfall and water temperature.

**Figure 11:**
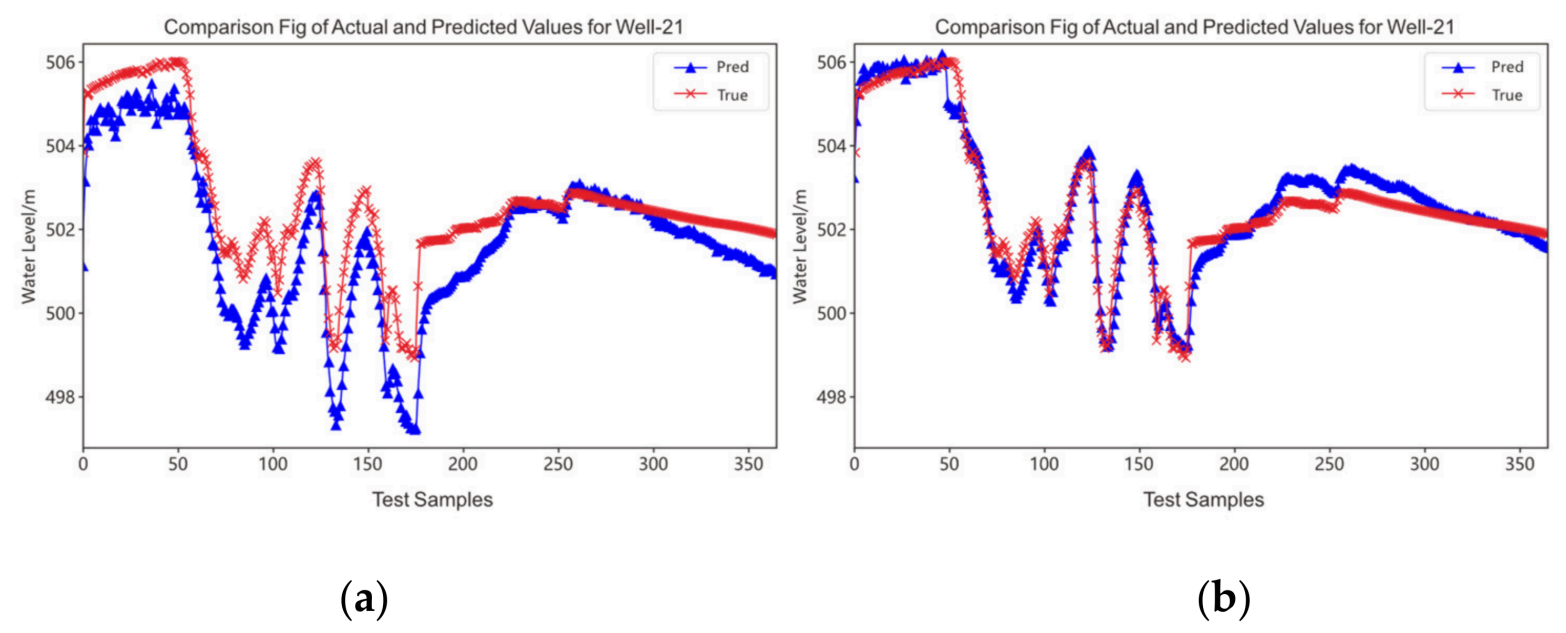
Prediction results for well-21 under two input settings. (**a**) Spatial features + groundwater level. (**b**) Spatial features + groundwater level + rainfall and water temperature.

**Figure 12:**
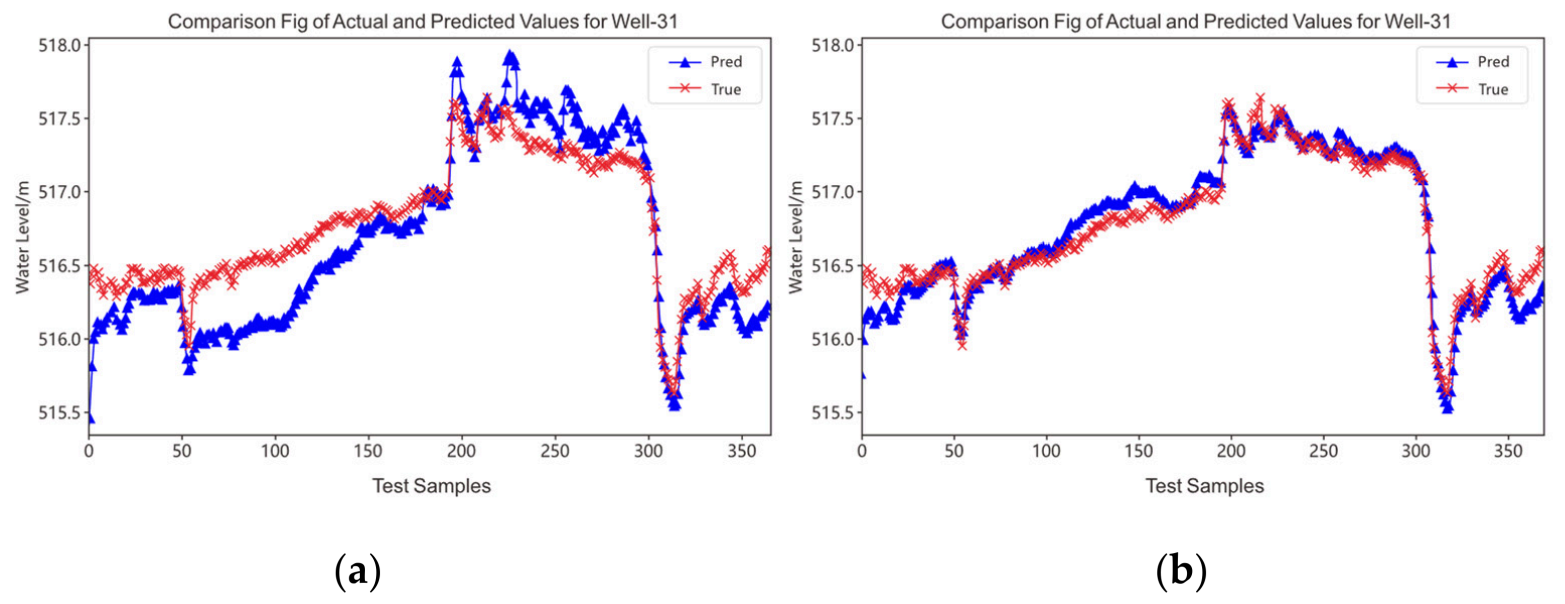
Prediction results for well-31 under two input settings. (**a**) Spatial features + groundwater level. (**b**) Spatial features + groundwater level + rainfall and water temperature.

**Figure 13:**
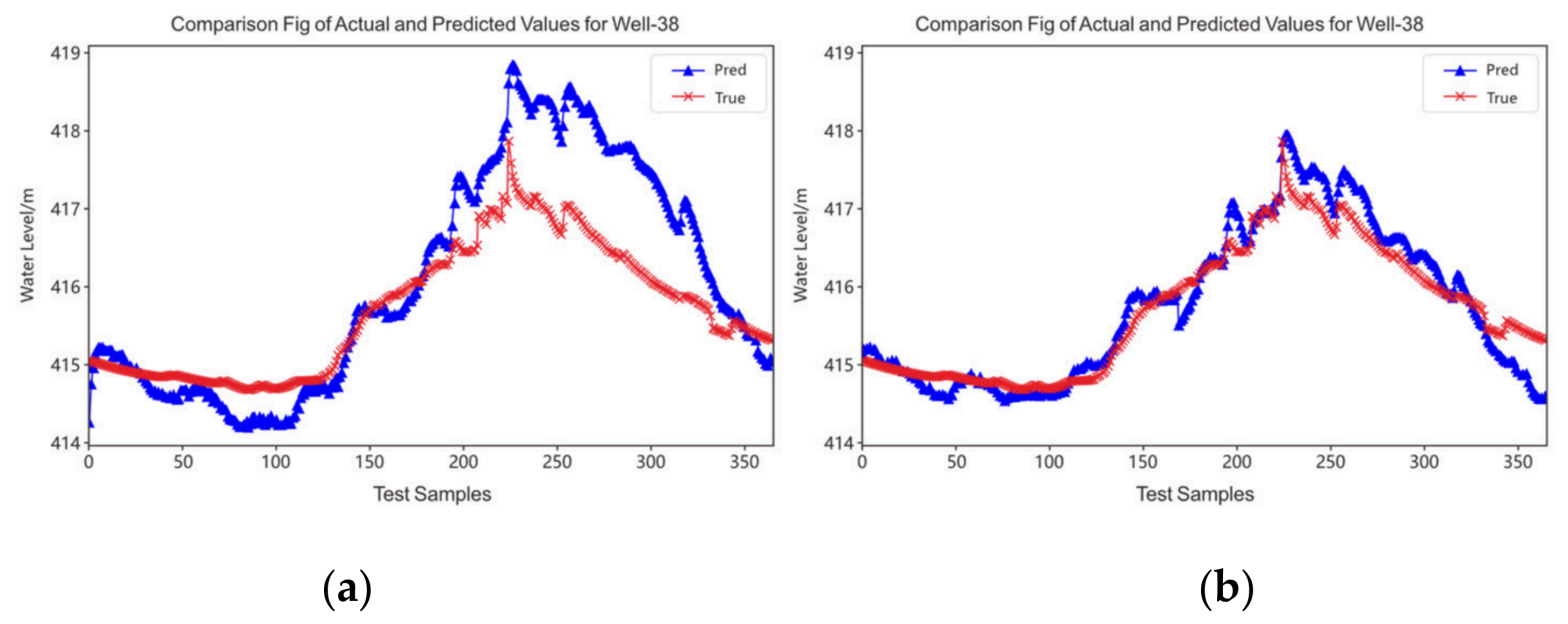
Prediction results for well-38 under two input settings. (**a**) Spatial features + groundwater level. (**b**) Spatial features + groundwater level + rainfall and water temperature.

**Figure 14:**
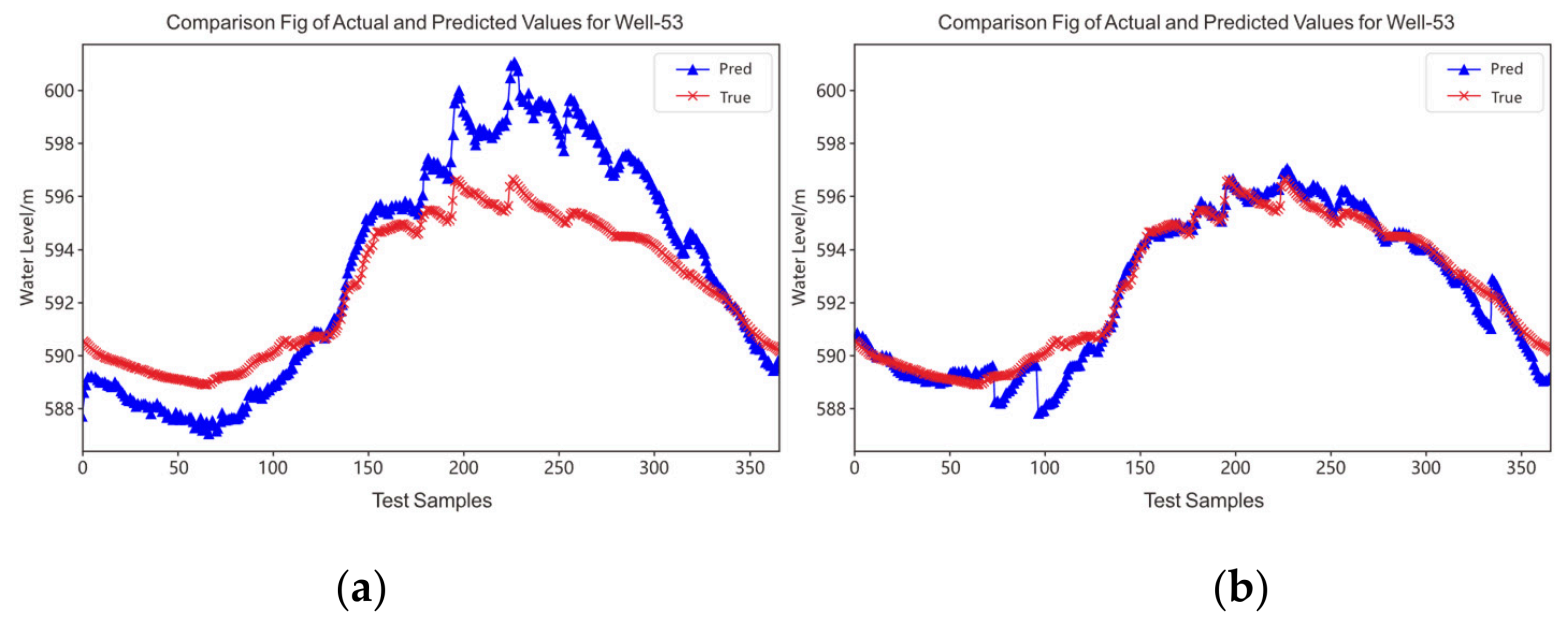
Prediction results for well-53 under two input settings. (**a**) Spatial features + groundwater level. (**b**) Spatial features + groundwater level + rainfall and water temperature.

Groundwater level prediction was evaluated using data from 56 observation wells, and the last 366 samples were used for testing. Two forecasting scenarios were compared. The first used only spatial features and historical groundwater level data, whereas the second additionally incorporated water temperature and rainfall. Model performance was assessed using the coefficient of determination (R^2^), mean absolute error (MAE), and root mean square error (RMSE). All evaluation metrics under the two input settings were calculated using the same test target samples for each monitoring well to ensure a direct and consistent comparison. Overall, the GCN-LSTM model performed well under both scenarios, indicating that the proposed framework can provide stable regional groundwater level forecasts even when the available input information is limited. The multi-factor setting yielded lower MAE and RMSE and higher R^2^ for most monitoring wells, indicating improved performance across the monitoring network. Table 4 summarizes the prediction performance for the first 10 monitoring wells, while the complete results for all 56 wells are provided in Table A3 of Appendix A.3. MAE and RMSE were calculated on normalized groundwater level values. For visualization in Figure 9, Figure 10, Figure 11, Figure 12, Figure 13 and Figure 14, the predicted and observed groundwater levels were inverse-transformed to their original physical scale (m).

To further illustrate the predictive behavior of the model under the two scenarios, six representative wells, namely well-1, well-10, well-21, well-31, well-38, and well-53, were selected for visual comparison. Figure 9, Figure 10, Figure 11, Figure 12, Figure 13 and Figure 14 present the predicted and observed groundwater levels for these wells under the scenario using only spatial and groundwater level features and the scenario using spatial features together with three temporal variables. In both cases, the predicted series generally follow the observed temporal trends, indicating that the proposed framework is capable of capturing the main evolution pattern of groundwater fluctuations. Nevertheless, the scenario with additional temporal variables shows a closer fit to local fluctuations, especially around peaks, troughs, and abrupt changes.

Taken together, the quantitative results in Table 4 and the visual comparisons in Figure 9, Figure 10, Figure 11, Figure 12, Figure 13 and Figure 14 indicate that incorporating rainfall and water temperature generally improves forecasting performance, although the extent of improvement varies among wells. Well-10 illustrates a pronounced limitation of the S+W setting: predictions substantially overestimate the observed groundwater levels during the latter part of the testing period, whereas the S+T predictions follow the observations more closely (Figure 10). This contrast highlights the limitations of the reduced-input configuration at this well. To further isolate the contribution of graph-based spatial modeling, a controlled network-wide comparison with a conventional LSTM baseline is presented in the following section.

### 3.6. Network-Wide Comparison with the LSTM Baseline

The network-wide comparison between GCN-LSTM and the conventional LSTM followed the controlled S+W protocol described in Section 3.2.

**Figure 15:**
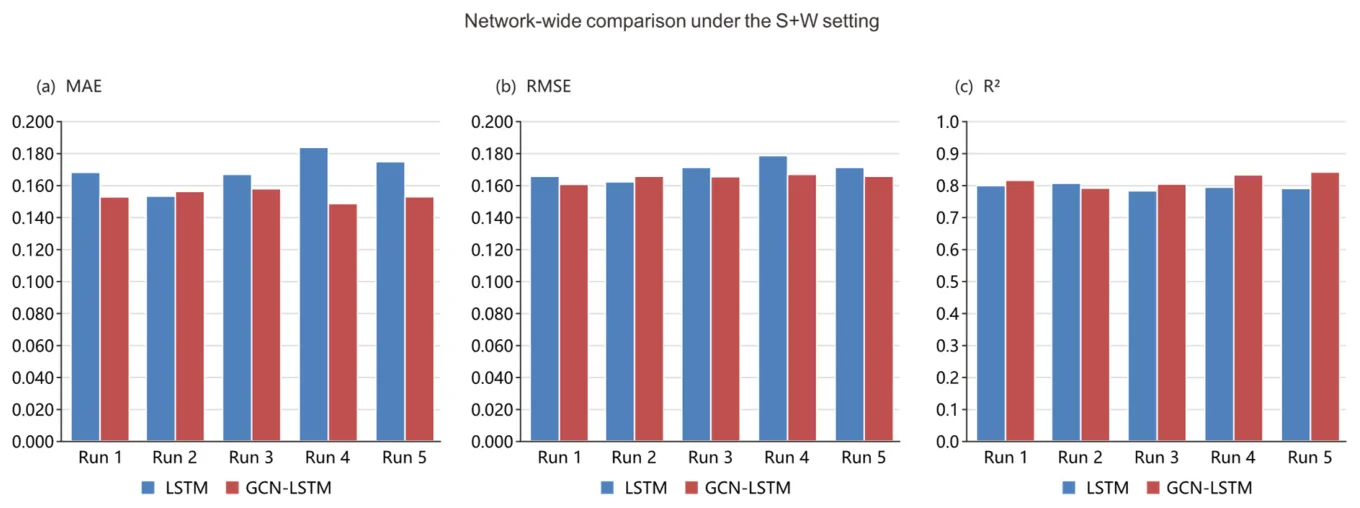
Network-wide comparison between GCN-LSTM and LSTM under the S + W setting across five independent runs: (**a**) MAE, (**b**) RMSE, and (**c**) R^2^.

Both models were independently trained using five random seeds. For each seed, MAE, RMSE, and R^2^ were calculated for individual wells and then averaged across the 56-well monitoring network. The results are shown in Figure 15 and Table 5.

As shown in Figure 15 and Table 5, GCN-LSTM achieved better average performance than LSTM over the five independent runs. The mean MAE decreased from 0.17000 ± 0.01123 to 0.15432 ± 0.00331, while the mean RMSE decreased from 0.17810 ± 0.00656 to 0.17271 ± 0.00250, corresponding to reductions of approximately 9.22% and 3.02%, respectively. The mean R^2^ increased from 0.83353 ± 0.00934 to 0.85620 ± 0.02213. Values are reported as the mean ± sample standard deviation across the five runs. GCN-LSTM outperformed LSTM on all three metrics in four of the five runs, although its R^2^ exhibited greater variation across runs.

These results support the predictive benefit of incorporating graph-based inter-well information alongside temporal modeling under the evaluated S+W setting. The improvement concerns average network-level performance and does not imply uniform gains across individual runs or monitoring wells.

### 3.7. Supplementary Comparison with Single-Well Forecasting Models

In addition to the network-wide controlled comparison presented in Section 3.6, a supplementary comparison was conducted for well-56 using LSTM, GRU, STA-LSTM, and GCN-LSTM. The LSTM, GRU, and STA-LSTM baseline models were adopted with reference to our previous groundwater level forecasting study [48]. The corresponding baseline results reported here were obtained from previous single-well experiments on the Chengdu dataset using well-56 as the common prediction target, allowing a supplementary comparison with GCN-LSTM at the same monitoring location. This comparison is intended as an illustrative single-well benchmark rather than a comprehensive evaluation across all 56 monitoring wells.

**Figure 16:**
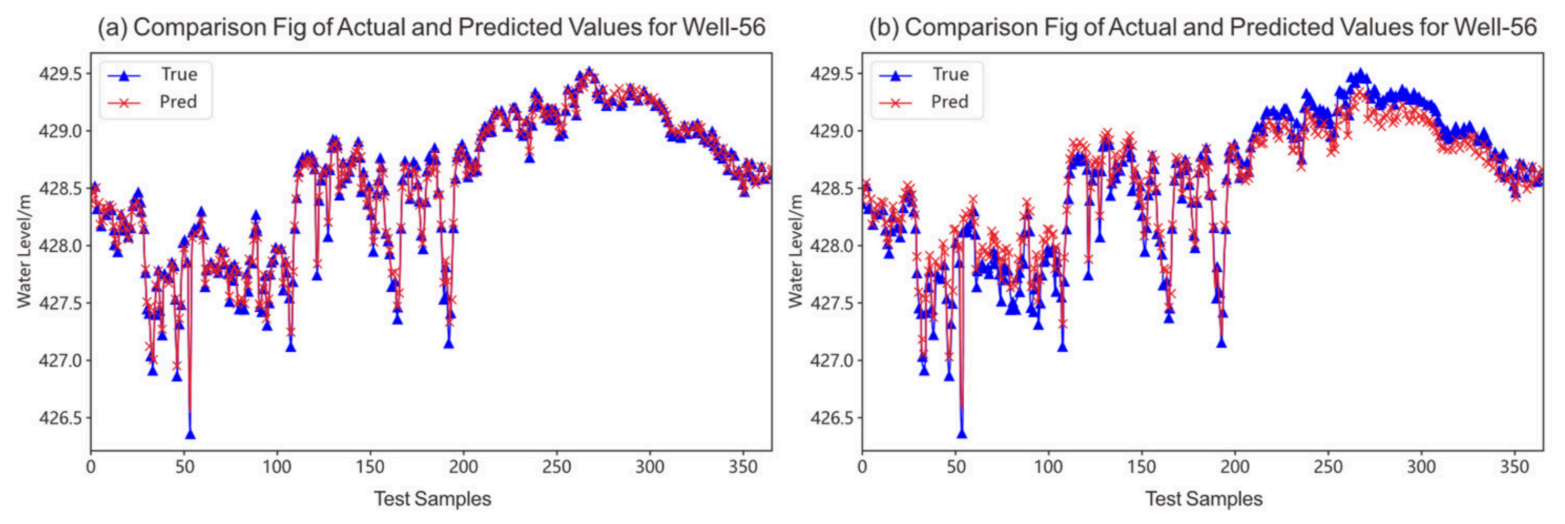
Forecasting results for well-56: (**a**) GCN-LSTM and (**b**) STA-LSTM.

The quantitative results are summarized in Table 6. GCN-LSTM achieved the lowest RMSE and the highest R^2^ among the compared models. Compared with STA-LSTM, the MAE decreased slightly from 0.02437 to 0.02415, while the RMSE decreased from 0.02946 to 0.02568 and R^2^ increased from 0.96673 to 0.97472. Figure 16 further compares the observed and predicted groundwater levels during the testing period. Both STA-LSTM and GCN-LSTM captured the overall temporal pattern, whereas GCN-LSTM followed several local fluctuations more closely.

These results suggest that inter-well spatial information may provide complementary predictive value for well-56. However, because the comparison was limited to one monitoring location, the findings should not be generalized to all wells.

## 4. Discussion

The results indicate that the proposed GCN-LSTM framework is able to provide reliable groundwater level forecasts under both input settings, namely the scenario using only spatial features and groundwater level data and the scenario that further includes rainfall and water temperature. This suggests that the framework has the capacity to learn both spatial dependence among wells and temporal variations in groundwater level sequences, even when the amount of available input information is limited. From a practical perspective, the S+W setting is relevant to existing monitoring networks where rainfall and water-temperature inputs are unavailable. This comparison concerns the availability of additional input variables, while the number and spatial distribution of monitoring wells remain unchanged.

A clear performance improvement is observed when rainfall and water temperature are incorporated. This result is hydrologically reasonable because groundwater fluctuations are not governed only by the historical evolution of groundwater levels, but are also influenced by external hydro-meteorological drivers. Rainfall provides direct information on recharge and infiltration conditions, while water temperature reflects seasonal variability and environmental changes that may be associated with groundwater response. By incorporating these additional temporal variables, the model becomes more capable of capturing fluctuation trends and identifying turning points in groundwater levels. The stronger performance in the multi-factor scenario therefore suggests that regional groundwater forecasting benefits from the integration of both groundwater observations and relevant environmental drivers.

Another important finding is the contribution of inter-well spatial modeling to regional prediction performance. The network-wide controlled comparison showed that GCN-LSTM achieved lower MAE and RMSE values and a higher R^2^ than the conventional LSTM under the same S+W setting. Because both models were trained and evaluated using the same groundwater level inputs, temporal window, data split, and optimization settings, the improved performance indicates that the graph-based representation provides predictive information beyond the temporal dependencies captured by LSTM alone. In a multi-well groundwater system, groundwater dynamics at an individual well may be related to conditions at other monitoring locations through spatial proximity and shared hydrogeological characteristics. The graph representation provides a structured mechanism for incorporating these relationships, while the GCN component aggregates spatially related information before temporal dependencies are modeled by the LSTM. This is particularly relevant for urban groundwater networks, where wells are often distributed irregularly and their interactions cannot be adequately represented by conventional sequence models that treat each well independently.

The supplementary comparison for well-56 is consistent with the network-wide findings. GCN-LSTM achieved a lower RMSE and a higher R^2^ than the single-well forecasting models considered in this benchmark. The results suggest that information from surrounding wells may provide complementary predictive signals for local groundwater level forecasting. However, the difference in MAE between GCN-LSTM and STA-LSTM is small, indicating that the main improvement is associated with reducing relatively large prediction errors rather than uniformly improving all predictions.

Although the proposed framework shows promising results, several limitations should be noted. First, the present study is based on a single urban case in Chengdu, and the transferability of the framework to other groundwater systems with different hydrogeological settings still needs to be examined. Second, the graph structure is constructed from predefined spatial and attribute similarity assumptions. While these assumptions are reasonable for the current dataset, alternative graph construction strategies may produce different results and should be explored in future work. Third, missing values, noisy observations, and abnormal measurements are handled through preprocessing before model training. A unified framework that can jointly address data quality issues and forecasting may further improve robustness in practical applications. Fourth, although the network-wide comparison with LSTM provides controlled evidence for the contribution of graph-based spatial modeling, the present ablation focuses on the S+W setting. Further studies could extend the comparison to additional input configurations and alternative spatial graph formulations. Finally, all 56 monitoring wells were included during training, and testing evaluated subsequent groundwater levels at these same wells. The reported results therefore do not establish generalization to withheld wells or performance under reduced spatial monitoring coverage. A leave-some-wells-out evaluation is needed to assess these capabilities in future work.

Overall, the results demonstrate that regional groundwater forecasting can benefit from combining graph-based spatial representation with temporal sequence learning. The controlled comparison with the LSTM baseline further supports the complementary role of inter-well spatial information in the proposed framework. The proposed framework is particularly suitable for monitoring networks with irregular well layouts and varying levels of data availability. In this sense, the study provides not only a prediction model for the Chengdu case, but also a general modeling perspective for regional groundwater level forecasting in urban monitoring systems.

## 5. Conclusions

This study addressed the problem of multi-well groundwater level forecasting within regional groundwater monitoring networks by developing a GCN-LSTM framework that jointly models inter-well spatial dependence and temporal groundwater dynamics. Using daily observations from 56 monitoring wells in Chengdu, two forecasting scenarios were examined, including a setting based on spatial features and historical groundwater levels only, and a setting further incorporating rainfall and water temperature. The results show that the proposed framework can provide reliable and stable groundwater level forecasts for multiple wells under both scenarios. In particular, the scenario with additional temporal drivers achieved better performance, indicating that the integration of hydro-meteorological information improves the model’s ability to capture local fluctuations and turning points in groundwater level dynamics.

The additional network-wide comparison with the conventional LSTM provides controlled evidence supporting the predictive benefit of graph-based spatial modeling under the S+W setting. Averaged over five independent runs, GCN-LSTM achieved lower network-average MAEs and RMSEs and a higher network-average R^2^ across the 56-well monitoring network. These findings support the value of incorporating inter-well spatial information alongside temporal modeling, although the improvements were not uniform across runs. Together with the comparison between the S+W and S+T scenarios, the results suggest that both graph-based spatial modeling and the inclusion of additional hydro-meteorological inputs can improve regional groundwater level forecasting under the conditions evaluated in this study.

Several limitations should also be noted. The present study was validated using one urban case in Chengdu, and the transferability of the framework to other hydrogeological settings remains to be further examined. In addition, the graph structure depends on predefined spatial and attribute similarity assumptions, and missing or noisy observations are handled through preprocessing rather than being modeled jointly with forecasting. Future work will therefore focus on evaluating the framework across different groundwater systems, exploring alternative graph construction strategies, and improving the treatment of data quality issues in regional groundwater prediction.

## Figures and Tables

**Table 1:** Statistical Table of Spatial Features of Observation Wells.

Well ID	Longitude	Latitude	Wellhead Elevation (m)	Well Depth (m)
well-1	104.1165540	30.63808000	492.815	16.0
well-2	104.0097128	30.66046697	501.620	12.8
well-3	104.0807823	30.67020439	496.260	25.0
well-4	103.9860347	30.70459650	512.320	40.0
well-5	104.0572905	30.69116975	499.190	30.0
well-6	104.0115476	30.70619214	511.490	30.0
well-7	104.0232957	30.72968492	511.890	30.0
well-8	104.0744763	30.62929447	489.720	20.0
well-9	104.2055740	30.57715600	527.638	103.0
well-10	104.3358723	30.87778258	466.930	20.5

**Table 2:** Statistical Summary of Groundwater Level Records for Representative Observation Wells.

Statistic	well-7	well-17	well-24	well-42	well-52
Max(m)	511.810	522.092	507.535	685.169	492.637
Min(m)	498.332	511.932	495.017	675.157	480.732
Mean(m)	504.280	516.848	504.044	680.103	489.027
Range(m)	13.478	10.160	12.518	10.012	11.905
Std. Dev.(m)	2.688	2.263	3.440	1.844	1.502
Skewness	−0.161	−0.298	−1.452	−0.079	−1.469
Kurtosis	0.148	−0.844	0.988	−0.246	5.376

**Table 3:** Main specifications of the liquid-level monitoring sensor and RTU.

Component	Parameter	Specification
Liquid-level sensor	Sensor type	Submersible hydrostatic liquid-level transmitter
Liquid-level sensor	Measurement range	0−15 m
Liquid-level sensor	Accuracy	0.1%
Liquid-level sensor	Communication	RS485/Modbus
Liquid-level sensor	Power supply	9–36 VDC, with 12/24 VDC as nominal supply options
Liquid-level sensor	Protection rating	IP68
Liquid-level sensor	Diaphragm material	316L stainless steel
Liquid-level sensor	Installation method	Direct submersion
Liquid-level sensor	Environmental temperature	−20 °C to 65 °C
Liquid-level sensor	Medium temperature	−20 °C to 85 °C
RTU	Serial interfaces	RS485×1, RS232×1
RTU	Baud rate	2400–115,200 bps
RTU	Network	LTE-FDD, LTE-TDD
RTU	Voltage	8.4–16 V
RTU	Data transmission	MQTT to OneNET; HTTP platform retrieval
RTU	Transmission strategy	Configurable/adaptive transmission frequency

**Table 4:** Prediction Performance Comparison of GCN-LSTM Under Two Input Settings (Abbreviations: S + W—Spatial & Water Level only, S + T—Spatial & Temporal Features).

Well ID	Input Setting	MAE	RMSE	R^2^
well-1	S + W	0.10648	0.11584	0.79623
	S + T	0.07943	0.08263	0.89632
well-2	S + W	0.19323	0.19790	0.76341
	S + T	0.10346	0.11038	0.9264
well-3	S + W	0.22647	0.23416	0.80480
	S + T	0.17373	0.18237	0.88160
well-4	S + W	0.19968	0.23057	0.79074
	S + T	0.14374	0.16363	0.89461
well-5	S + W	0.18167	0.18984	0.81884
	S + T	0.13236	0.14363	0.89630
well-6	S + W	0.14542	0.15902	0.82312
	S + T	0.11937	0.12368	0.89300
well-7	S + W	0.26853	0.27706	0.76061
	S + T	0.22562	0.22900	0.83646
well-8	S + W	0.15426	0.1775	0.85296
	S + T	0.11739	0.12360	0.92870
well-9	S + W	0.32179	0.35387	0.72786
	S + T	0.23550	0.27370	0.83720
well-10	S + W	1.39687	1.76605	−14.36084
	S + T	0.08129	0.09320	0.95722

**Table 5:** Network-wide comparison between GCN-LSTM and LSTM under the S+W setting.

Run	Model	MAE	RMSE	R^2^
Run1	LSTM	0.168845114	0.173856654	0.841268938
	GCN-LSTM	0.153649710	0.168402087	0.851285543
Run2	LSTM	0.153792177	0.170144167	0.843682744
	GCN-LSTM	0.156405202	0.173521188	0.828975106
Run3	LSTM	0.167456571	0.179531879	0.820799031
	GCN-LSTM	0.158280261	0.172849066	0.843634638
Run4	LSTM	0.184206516	0.187425417	0.833277733
	GCN-LSTM	0.149514539	0.174668463	0.874274942
Run5	LSTM	0.175702390	0.179524815	0.828629113
	GCN-LSTM	0.153755764	0.174126834	0.882827713

**Table 6:** Supplementary comparison of forecasting models for well-56.

Model	MAE	RMSE	R^2^
LSTM	0.04547	0.04839	0.91023
GRU	0.04706	0.04985	0.90473
STA-LSTM	0.02437	0.02946	0.96673
GCN-LSTM	0.02415	0.02568	0.97472

## Data Availability

The data used in this study were obtained from the China Institute of Geological Environment Monitoring. The data supporting the findings of this study are available from the corresponding author upon reasonable request, subject to the applicable restrictions of the data provider.

## Abbreviations

The following abbreviations are used in this manuscript:

S + W	Spatial & Water Level only
S + T	Spatial & Temporal Features

## Appendix A

### Appendix A.1

Table A1 provides the complete statistical information of the spatial features for all 56 observation wells used in this study. The spatial attributes include longitude, latitude, wellhead elevation, and well depth, which characterize the geographic distribution and vertical characteristics of the groundwater monitoring network. These attributes were used to construct the spatial and attribute similarity matrices for graph representation in the proposed GCN-LSTM framework. The complete table is provided in the Appendix to ensure transparency and facilitate reproducibility of the graph construction process.

**Table A1:** Statistical Table of Spatial Features of Observation Wells.

Observation Well ID	Longitude	Latitude	Wellhead Elevation	Well Depth
well-1	104.116554	30.63808	492.815	16
well-2	104.0097128	30.66046697	501.62	12.8
well-3	104.0807823	30.67020439	496.26	25
well-4	103.9860347	30.7045965	512.32	40
well-5	104.0572905	30.69116975	499.19	30
well-6	104.0115476	30.70619214	511.49	30
well-7	104.0232957	30.72968492	511.89	30
well-8	104.0744763	30.62929447	489.72	20
well-9	104.205574	30.577156	527.638	103
well-10	104.3358723	30.87778258	466.93	20.5
well-11	104.3190949	30.82034986	460.47	20
well-12	104.051186	30.949104	526.868	20
well-13	104.1819494	30.84714128	445.165	20
well-14	104.1059697	30.81420072	461.242	20.2
well-15	104.0354556	30.86651886	484.598	20
well-16	103.8762191	30.75820803	547.79	30
well-17	103.9100337	30.69498394	523.62	20.3
well-18	104.689255	30.642672	454.452	100.6
well-19	104.4457128	30.83443019	472.03	11
well-20	104.4453138	30.84871072	443.03	11.5
well-21	103.8660155	30.61529381	506.82	20.5
well-22	103.9232631	30.62217572	505.29	30
well-23	103.8281016	30.56567294	496.53	30
well-24	103.8950699	30.63526619	509.37	30
well-25	103.8494111	30.57080097	495.13	20
well-26	103.839195	30.52712978	484.58	30
well-27	103.9334966	30.75874178	532.3	100
well-28	103.9592338	30.78032617	533.51	30
well-29	103.9651395	30.83696203	540.2	30
well-30	103.9730263	30.80710328	532.22	30
well-31	104.0093868	30.766494	518.87	38
well-32	103.9848816	30.76145586	524.68	36
well-33	103.6378706	30.50429856	456.423	30
well-34	103.4611544	30.54697761	508.499	30
well-35	103.5205722	30.50183853	477.404	100
well-36	103.7787169	30.41216531	417.596	20
well-37	103.8122483	30.48294486	430.145	20
well-38	103.8457419	30.44215781	420.784	20
well-39	103.6398092	30.84121097	592.283	100
well-40	103.7229158	30.91298008	605.342	30
well-41	103.5881956	30.94613903	715.4	21.2
well-42	103.8702278	31.04217222	692.13	30.2
well-43	103.9310917	30.94148333	574.27	30.1
well-44	104.1059222	30.96778889	513.23	30.2
well-45	103.8060861	31.04365	653.05	30.1
well-46	103.5819325	30.38734858	441.589	30
well-47	103.55301	30.39726539	445.572	30
well-48	103.5993201	30.66415747	552.03	100
well-49	103.6348944	30.73898497	537.974	30.1
well-50	103.6011506	30.55118006	473.237	30.1
well-51	103.6792786	30.74728425	538.373	30
well-52	103.6061533	30.60691528	493.519	29.9
well-53	103.5083233	30.74082589	602.818	30
well-54	103.5790256	30.60528569	495.28	29.9
well-55	104.45184	30.53633	440.787	86.5
well-56	104.335236	30.400795	452.178	83.8

### Appendix A.2

Table A2 presents the complete statistical summary of groundwater-level observations from all 56 monitoring wells during the study period. The table reports the main statistical characteristics of each well, including the mean value, standard deviation, minimum, maximum, and variation range of groundwater levels. These statistical indicators provide an overview of the temporal variability and heterogeneity among different monitoring locations within the regional groundwater network. The complete statistical information is provided to characterize the groundwater-level distribution and support the interpretation of forecasting performance across multiple wells.

**Table A2:** Statistical Table of Observation Wells.

Well ID	Max (m)	Min (m)	Mean (m)	Range (m)	Std. Dev.(m)	Skewness	Kurtosis
well-1	487.027	483.07	484.383	3.957	0.68	1.386	2.357
well-2	496.675	491.661	494.043	5.014	1.288	0.211	−0.641
well-3	493.013	489.683	490.686	3.33	0.686	0.529	−0.305
well-4	509.986	504.308	506.969	5.678	1.516	−1.097	−0.603
well-5	495.273	490.604	492.229	4.669	0.834	−0.305	−0.212
well-6	502.726	498.428	500.318	4.298	1.112	0.328	−0.669
well-7	511.81	498.332	504.28	13.478	2.688	−0.161	0.148
well-8	487.135	480.791	484.087	6.344	1.438	−0.737	−0.404
well-9	513.853	507.072	511.415	6.781	1.48	−0.854	0.21
well-10	461.243	456.899	458.034	4.344	0.828	1.654	2.684
well-11	458.83	456.577	457.762	2.253	0.365	−0.641	1.345
well-12	526.858	525.938	526.345	0.92	0.156	1.7	3.455
well-13	444.502	442.031	443.137	2.471	0.652	0.017	−1.096
well-14	458.381	449.351	455.224	9.03	1.491	−1.428	2.495
well-15	485.596	481.316	482.588	4.28	0.59	0.237	−0.077
well-16	543.042	539.492	541.677	3.55	0.567	−1.63	3.273
well-17	522.092	511.932	516.848	10.16	2.263	−0.298	−0.844
well-18	453.612	451.931	452.93	1.681	0.322	−0.391	−0.106
well-19	469.263	467.353	468.143	1.91	0.239	1.139	1.558
well-20	436.933	433.452	434.391	3.481	0.554	1.962	6.974
well-21	505.992	498.92	502.013	7.072	0.956	1.674	5.357
well-22	501.058	494.587	497.311	6.471	1.421	0.516	0.109
well-23	490.575	486.141	487.477	4.434	0.953	0.748	−0.059
well-24	507.535	495.017	504.044	12.518	3.44	−1.452	0.988
well-25	493.076	488.891	491.128	4.185	0.713	−1.479	2.451
well-26	483.464	476.627	480.263	6.837	1.419	−0.538	−0.343
well-27	530.989	527.06	528.782	3.929	0.672	−0.507	0.211
well-28	534.176	528.755	529.992	5.421	1.038	2.279	5.47
well-29	538.216	534.651	536.069	3.565	0.749	0.682	−0.257
well-30	530.96	527.222	529.464	3.738	0.738	−0.94	−0.478
well-31	517.676	515.635	516.645	2.041	0.349	0.218	−0.304
well-32	521.38	516.21	519.157	5.17	1.39	−0.433	−0.976
well-33	454.57	451.77	452.882	2.8	0.596	0.266	−0.749
well-34	507.601	505.597	506.948	2.004	0.358	−1.398	2.705
well-35	469.488	464.807	466.56	4.681	0.798	0.666	0.401
well-36	415.919	410.164	414.007	5.755	1.084	−2.029	4.366
well-37	429.168	420.743	426.138	8.425	2.053	−1.265	0.201
well-38	419.316	414.556	416.027	4.76	1.023	0.676	−0.433
well-39	591.211	587.698	589.513	3.513	0.526	−0.383	0.722
well-40	602.5	600.278	601.182	2.222	0.39	0.216	0.092
well-41	713.315	706.768	710.38	6.547	1.826	−0.121	−1.072
well-42	685.169	675.157	680.103	10.012	1.844	−0.079	−0.246
well-43	574.179	569.598	571.1	4.581	0.596	0.221	1.388
well-44	511.633	509.363	509.927	2.27	0.367	1.048	1.345
well-45	649.881	647.88	648.527	2.001	0.306	−0.197	−0.219
well-46	439.539	436.51	437.24	3.029	0.425	1.104	2.847
well-47	445.142	441.506	442.867	3.636	0.952	1.031	0.47
well-48	548.82	546.467	547.586	2.353	0.422	0.116	−0.257
well-49	534.358	532.401	533.166	1.957	0.382	0.292	−0.627
well-50	471.867	469.438	470.334	2.429	0.571	0.279	−1.095
well-51	537.119	534.918	536.031	2.201	0.433	0.128	−0.388
well-52	492.637	480.732	489.027	11.905	1.502	−1.469	5.376
well-53	598.726	588.896	593.027	9.83	2.244	−0.22	−1.251
well-54	492.109	485.689	487.68	6.42	1.305	0.397	−0.372
well-55	430.733	422.544	428.329	8.189	1.054	−1.392	4.516
well-56	431.371	426.362	429.422	5.009	0.853	−0.08	−0.103

### Appendix A.3

Table A3 provides the complete prediction performance comparison of the proposed GCN-LSTM framework across all 56 groundwater monitoring wells under the two input settings. The two scenarios include a reduced-input setting using historical groundwater observations and spatial features, and a multi-factor setting incorporating additional rainfall and water temperature variables. The forecasting performance is evaluated using mean absolute error (MAE), root mean square error (RMSE), and coefficient of determination (R^2^). The complete well-by-well results are provided to demonstrate the consistency of model performance across the regional monitoring network and to support the detailed analysis presented in the main text.

**Table A3:** Prediction Performance Comparison of GCN-LSTM Under Two Input Settings.

Well ID	Input Setting	MAE	RMSE	R^2^
well-1	S + W	0.10648	0.11584	0.79623
	S + T	0.07943	0.08263	0.89632
well-2	S + W	0.19323	0.1979	0.76341
	S + T	0.10346	0.11038	0.92640
well-3	S + W	0.22647	0.23416	0.80480
	S + T	0.17373	0.18237	0.88160
well-4	S + W	0.19968	0.23057	0.79074
	S + T	0.14374	0.16363	0.89461
well-5	S + W	0.18167	0.18984	0.81884
	S + T	0.13236	0.14363	0.89630
well-6	S + W	0.14542	0.15902	0.82312
	S + T	0.11937	0.12368	0.89300
well-7	S + W	0.26853	0.27706	0.76061
	S + T	0.22562	0.22900	0.83646
well-8	S + W	0.15426	0.17750	0.85296
	S + T	0.11739	0.12360	0.92870
well-9	S + W	0.32179	0.35387	0.72786
	S + T	0.23550	0.27370	0.83720
well-10	S + W	1.39687	1.76605	-14.36084
	S + T	0.08129	0.09320	0.95722
well-11	S + W	0.23287	0.28279	0.77769
	S + T	0.17236	0.23710	0.84372
well-12	S + W	0.26295	0.26864	0.77623
	S + T	0.16360	0.18372	0.89534
well-13	S + W	0.13579	0.14352	0.92197
	S + T	0.07237	0.09821	0.96346
well-14	S + W	0.24198	0.26502	0.79068
	S + T	0.21250	0.23700	0.83260
well-15	S + W	0.14931	0.17619	0.87864
	S + T	0.09920	0.11782	0.94573
well-16	S + W	0.13704	0.15260	0.85884
	S + T	0.07150	0.09323	0.94731
well-17	S + W	0.17906	0.26278	0.87636
	S + T	0.16327	0.28347	0.85612
well-18	S + W	0.17164	0.17853	0.81565
	S + T	0.12231	0.12932	0.90327
well-19	S + W	0.14071	0.16947	0.84767
	S + T	0.06170	0.09342	0.95371
well-20	S + W	0.09385	0.09747	0.93371
	S + T	0.07390	0.07832	0.95720
well-21	S + W	0.08993	0.09218	0.93402
	S + T	0.07352	0.07631	0.95478
well-22	S + W	0.13825	0.14982	0.82540
	S + T	0.05832	0.07017	0.96170
well-23	S + W	0.14218	0.23349	0.83597
	S + T	0.16712	0.21362	0.86270
well-24	S + W	0.13947	0.15994	0.90224
	S + T	0.12613	0.13221	0.93320
well-25	S + W	0.12370	0.13214	0.89258
	S + T	0.06362	0.07823	0.96235
well-26	S + W	0.16128	0.17942	0.86466
	S + T	0.07320	0.09320	0.96348
well-27	S + W	0.22194	0.23801	0.86227
	S + T	0.11289	0.19843	0.90427
well-28	S + W	0.12143	0.12991	0.90926
	S + T	0.07289	0.08710	0.95921
well-29	S + W	0.12569	0.15531	0.93366
	S + T	0.08782	0.09680	0.97423
well-30	S + W	0.13289	0.16943	0.86100
	S + T	0.09732	0.09830	0.95321
well-31	S + W	0.12804	0.17431	0.87308
	S + T	0.09623	0.10632	0.95278
well-32	S + W	0.10042	0.12386	0.90154
	S + T	0.08516	0.08678	0.95167
well-33	S + W	0.10406	0.11218	0.82873
	S + T	0.08340	0.08430	0.90328
well-34	S + W	0.14620	0.15317	0.83338
	S + T	0.10910	0.11670	0.90328
well-35	S + W	0.14673	0.21937	0.90839
	S + T	0.08150	0.18900	0.93200
well-36	S + W	0.13790	0.14658	0.84040
	S + T	0.08670	0.09340	0.93520
well-37	S + W	0.13024	0.17459	0.85532
	S + T	0.09753	0.10540	0.94727
well-38	S + W	0.18459	0.19326	0.84517
	S + T	0.08340	0.12318	0.93710
well-39	S + W	0.14158	0.14624	0.82981
	S + T	0.07100	0.08510	0.94237
well-40	S + W	0.13371	0.14096	0.83183
	S + T	0.07567	0.08398	0.94031
well-41	S + W	0.08845	0.08946	0.93592
	S + T	0.07178	0.07678	0.95280
well-42	S + W	0.32750	0.33215	0.76705
	S + T	0.21678	0.26178	0.85530
well-43	S + W	0.13851	0.14209	0.82956
	S + T	0.08470	0.08930	0.93268
well-44	S + W	0.08637	0.08921	0.95154
	S + T	0.06330	0.07830	0.96267
well-45	S + W	0.09044	0.09361	0.94727
	S + T	0.06232	0.07890	0.96254
well-46	S + W	0.12549	0.14332	0.86256
	S + T	0.06890	0.08900	0.94700
well-47	S + W	0.12943	0.13389	0.85520
	S + T	0.08903	0.08950	0.93530
well-48	S + W	0.12189	0.12439	0.90661
	S + T	0.07126	0.08281	0.95861
well-49	S + W	0.10538	0.13280	0.88790
	S + T	0.05670	0.07156	0.96745
well-50	S + W	0.12479	0.12896	0.92148
	S + T	0.07832	0.09730	0.95530
well-51	S + W	0.11639	0.12398	0.86564
	S + T	0.06383	0.08110	0.94251
well-52	S + W	0.13178	0.15635	0.86883
	S + T	0.08930	0.09379	0.95280
well-53	S + W	0.12543	0.16320	0.88388
	S + T	0.07890	0.09780	0.95830
well-54	S + W	0.19942	0.21389	0.86469
	S + T	0.12687	0.13561	0.94561
well-55	S + W	0.09759	0.10337	0.91910
	S + T	0.06330	0.08320	0.94759
well-56	S + W	0.04364	0.04841	0.91016
	S + T	0.02415	0.02568	0.97472

### Appendix A.4

Table A4 provides the complete missing data statistics for groundwater level observations from all 56 monitoring wells before imputation. The table reports the total number of expected daily records, the number of missing observations, and the corresponding missing data percentage for each well over the 1826-day study period. In total, 20,809 of 102,256 expected groundwater level observations were missing, corresponding to an overall missing rate of 20.35%. The missing rate varied substantially among monitoring wells, ranging from 0.55% to 56.63%. These statistics characterize the spatial variability in data completeness across the monitoring network and provide additional transparency regarding the dataset prior to Random Forest-based missing value reconstruction.

**Table A4:** Missing Groundwater-Level Statistics for the 56 Monitoring Wells.

Well ID	Total Observations	Missing Values	Missing Rate (%)
well-1	1826	113	6.19
well-2	1826	732	40.09
well-3	1826	168	9.2
well-4	1826	713	39.05
well-5	1826	402	22.02
well-6	1826	399	21.85
well-7	1826	110	6.02
well-8	1826	361	19.77
well-9	1826	822	45.02
well-10	1826	226	12.38
well-11	1826	539	29.52
well-12	1826	216	11.83
well-13	1826	546	29.9
well-14	1826	733	40.14
well-15	1826	313	17.14
well-16	1826	290	15.88
well-17	1826	333	18.24
well-18	1826	27	1.48
well-19	1826	128	7.01
well-20	1826	1034	56.63
well-21	1826	328	17.96
well-22	1826	70	3.83
well-23	1826	101	5.53
well-24	1826	454	24.86
well-25	1826	458	25.08
well-26	1826	121	6.63
well-27	1826	208	11.39
well-28	1826	251	13.75
well-29	1826	284	15.55
well-30	1826	328	17.96
well-31	1826	191	10.46
well-32	1826	327	17.91
well-33	1826	273	14.95
well-34	1826	233	12.76
well-35	1826	347	19
well-36	1826	379	20.76
well-37	1826	203	11.12
well-38	1826	313	17.14
well-39	1826	314	17.2
well-40	1826	387	21.19
well-41	1826	725	39.7
well-42	1826	348	19.06
well-43	1826	238	13.03
well-44	1826	404	22.12
well-45	1826	746	40.85
well-46	1826	473	25.9
well-47	1826	505	27.66
well-48	1826	484	26.51
well-49	1826	649	35.54
well-50	1826	241	13.2
well-51	1826	447	24.48
well-52	1826	312	17.09
well-53	1826	366	20.04
well-54	1826	275	15.06
well-55	1826	811	44.41
well-56	1826	10	0.55
Overall	102,256	20,809	20.35
